# Predicting psychopathology symptom trajectories using machine learning: a 33‐year prospective study

**DOI:** 10.1111/jcpp.70180

**Published:** 2026-06-15

**Authors:** Seda Sacu, Fabian Streit, Stephanie H. Witt, Tobias Banaschewski, Nathalie E. Holz, Katharina Schultebraucks

**Affiliations:** ^1^ Department of Child and Adolescent Psychiatry and Psychotherapy Central Institute of Mental Health, Medical Faculty Mannheim, University of Heidelberg Mannheim Germany; ^2^ German Center for Mental Health (DZPG), partner site Mannheim‐Heidelberg‐Ulm Mannheim Germany; ^3^ Hector Institute for Artificial Intelligence in Psychiatry Central Institute of Mental Health, Medical Faculty Mannheim, University of Heidelberg Mannheim Germany; ^4^ Department of Psychiatry and Psychotherapy Central Institute of Mental Health, Medical Faculty Mannheim, Heidelberg University Mannheim Germany; ^5^ Department of Genetic Epidemiology in Psychiatry Central Institute of Mental Health, Medical Faculty Mannheim, University of Heidelberg Mannheim Germany; ^6^ Department of Psychiatry NYU Grossman School of Medicine New York NY USA; ^7^ Department of Population Health NYU Grossman School of Medicine New York NY USA

**Keywords:** Symptom trajectories, developmental psychopathology, growth mixture models, machine learning, internalizing symptoms, externalizing symptoms

## Abstract

**Background:**

While previous literature has established a link between psychopathology symptom trajectories and psychosocial risk, most studies have not extended into adulthood or incorporated comprehensive risk measures. We aimed to identify symptom trajectories for externalizing and internalizing symptoms over a 25‐year span and predict them using machine learning.

**Methods:**

We used data from a longitudinal birth cohort study (*N* = 317, 171 females). Psychopathology symptoms were measured between age 8 and 33. Symptom trajectories were identified using growth mixture models. An extensive battery of risk and protective measures over the lifespan (e.g., polygenic scores, psychosocial risk factors, temperament) was used to train machine learning‐based classification models.

**Results:**

We identified three trajectories for both externalizing and internalizing symptoms (entropy > 0.8, likelihood ratio test *p* < .05): Low, increasing (late‐adolescent emergence), and decreasing (childhood‐limited) symptoms classes. Both genetic (e.g., polygenic scores for cognition and mental disorders) and environmental factors (e.g., adverse family characteristics, poor social functioning) predicted symptom trajectories. For externalizing symptoms trajectories, random forest achieved fair discriminatory power with a multiclass macro‐average AUC of 0.77 and a multiclass micro‐average of 0.88. For internalizing symptoms trajectories, logistic regression achieved modest discriminatory power with a multiclass macro‐average AUC of 0.75 and a multiclass micro‐average of 0.83.

**Conclusions:**

Psychopathology risk trajectories vary across development and are influenced by diverse risk factors. Understanding these risk factors can support early identification of at‐risk individuals and inform targeted prevention and intervention efforts.

## Introduction

Mental health conditions are among the leading causes of disease burden (GBD 2019 Mental Disorders Collaborators, 2022), affecting millions of individuals worldwide (Freeman, 2022) and contributing to poor physical health (Scott et al., 2016) and long‐term economic consequences (Alaie, Svedberg, Ropponen, & Narusyte, 2023; Vergunst et al., 2023). Many of these conditions emerge early in life (McGrath et al., 2023), with 75% of mental disorders manifest before the age of 25 (McGorry & Mei, 2018) and nearly half of the population developing a condition by age 75 (McGrath et al., 2023). Given their widespread impact, addressing mental health through early diagnosis, effective treatment, and preventive interventions is essential for improving quality of life and reducing societal costs.

Symptom trajectories for internalizing and externalizing symptoms vary widely. While most individuals (~70%–80%) experience low symptoms levels throughout development (Fernandez Castelao & Kröner‐Herwig, 2014; Kemmis‐Riggs, Grove, McAloon, & Berle, 2020; Weeks et al., 2014), research has also identified distinct risk trajectories for psychopathology, including childhood‐limited (decreasing; Kemmis‐Riggs et al., 2020; Kjeldsen, Nes, Sanson, Ystrom, & Karevold, 2021; Nivard et al., 2017; Weeks et al., 2014), adolescent‐onset (increasing) (Kemmis‐Riggs et al., 2020; Kjeldsen et al., 2021; Nivard et al., 2017; Weeks et al., 2014), and stable high (Kjeldsen et al., 2021; Klein et al., 2019; Weeks et al., 2014). These trajectories were generally similar across symptom dimensions (Korhonen, Luoma, Salmelin, Siirtola, & Puura, 2018) and sexes (Fernandez Castelao & Kröner‐Herwig, 2014; Klinge, Warschburger, Busching, & Klein, 2023). Some studies additionally associated these risk trajectories with psychosocial risk factors, including early life adversities (Fernandez Castelao & Kröner‐Herwig, 2014; Kjeldsen et al., 2021; Klein et al., 2019; Klinge et al., 2023; Weeks et al., 2014), life stress (Fernandez Castelao & Kröner‐Herwig, 2014), parenting (Fernandez Castelao & Kröner‐Herwig, 2014), and temperament (Kjeldsen et al., 2021; Weeks et al., 2014). Internalizing symptoms trajectories are linked more strongly to peer victimization/rejection (Rappaport et al., 2021) and maternal mental health (Bevilacqua, Kelly, Heilmann, Priest, & Lacey, 2021), while harsh parenting (Bevilacqua et al., 2021; Wiggins, Mitchell, Hyde, & Monk, 2015), violence exposure (Taylor et al., 2018), and prenatal maternal smoking (Bista et al., 2024; Speyer et al., 2022) were stronger predictors of externalizing symptoms trajectories.

While previous literature has significantly advanced our understanding of developmental psychopathology trajectories and associated risk factors, several gaps remain. First, most studies have modeled symptom trajectories primarily during childhood and adolescence. Although these periods are critical for problem onset (McGrath et al., 2023), a considerable amount of individuals develop mental problems later in life, and symptom trajectories may show distinct growth patterns when adult symptomology is included. Additionally, few studies have compared the risk trajectories in terms of adulthood outcomes (Korhonen et al., 2018; Petersen, Bates, Dodge, Lansford, & Pettit, 2015). A comprehensive view of the enduring effects of risk trajectories can shed light on long‐term outcomes. Second, most studies have relied on a limited set of psychosocial measures while overlooking genetic factors that contribute to heterogeneous symptom trajectories. Notably, a limited number of studies have explored the role of genetic liability in the trajectories of internalizing and externalizing symptoms (Speyer et al., 2022). Incorporating a broader range of variables is essential for better characterization of risk groups. Lastly, previous studies solely used classical statistical approaches, such as multinomial regression, to distinguish the risk trajectories. Using more advanced methods, such as machine learning, will bring several advantages, including handling high‐dimensional data, capturing complex, non‐linear patterns, testing predictive accuracy, and identifying the most relevant features for each trajectory.

Machine learning approaches have recently gained popularity for predicting mental health outcomes. Previous studies in child and adolescent samples have used classification‐based machine learning methods to predict the presence of a wide range of mental health conditions, reporting fair to excellent model performance (Berluti, Amormino, Potter, Wshah, & Marsh, 2025; De Lacy, Ramshaw, & Lam, 2025; Pujadas et al., 2025; Tate et al., 2020). However, most of this work has focused on binary classification tasks (Berluti et al., 2025; De Lacy et al., 2025; Pujadas et al., 2025; Tate et al., 2020) and on outcomes assessed over relatively short follow‐up periods (Berluti et al., 2025; De Lacy et al., 2025). A recent study attempted to predict developmental symptom trajectories for externalizing and internalizing problems in children using machine learning approaches (Adhikari, You, Chen, Cheng, & Huang, 2023). The authors identified two trajectories for each domain (low vs. high risk). Prediction performance was fair for externalizing symptoms (AUC = 0.75–0.78) and poor for internalizing symptoms (AUC = 0.62–0.67).

The current study aimed to identify externalizing and internalizing symptoms trajectories from childhood to adulthood over 25 years in a deep‐phenotyped at‐risk cohort. Externalizing and internalizing symptoms were chosen to capture broad patterns of psychopathology. After identifying the trajectories, we tested if these trajectories could be predicted by a comprehensive list of prospectively collected environmental (e.g., childhood adversities, life stress) and biological (e.g., polygenic scores, hormones) measures. Based on previous literature, we expected to identify common risk trajectories, such as increasing, decreasing, or high‐stable, in addition to low symptoms. We also expected that machine learning models would predict the symptom trajectories with good discriminatory power. Additionally, we examined whether symptom trajectories were associated with adult functioning, lifespan stress exposure, coping skills, and resources.

## Methods

### Participants

The current study is conducted within the framework of the Mannheim Study of Children at Risk (Laucht et al., 2000). The initial sample included 384 infants recruited from obstetric and children's hospitals in Germany between 1986 and 1988. These participants were followed from their birth up to around 33 years across 11 assessment waves, with a final compliance rate of 66.7% (*N* = 256). For detailed sample descriptions and attrition bias analyses, see Appendix S1 (Tables S1–S4). Psychopathology measures were available from 8 years on across eight assessment waves (T4–T11). To reliably model symptom trajectories, we only included 317 participants (171 females, based on sex at birth) with available data from at least five assessment waves. The study was approved by the ethics committee of the University of Heidelberg and conducted in accordance with the Declaration of Helsinki. All participants gave written informed consent.

### Psychological measures

#### Psychopathology

For the childhood period (8–11 years), we measured internalizing and externalizing symptoms using the Child Behavior Checklist (Achenbach, 1991), which was filled by caregivers. Youth rated their own behavior during adolescence (15 years) via the youth self‐report (Achenbach, 1991). The parent‐youth agreement at age 15 was moderate, consistent with previous findings (Berg‐Nielsen, Vika, & Dahl, 2003; Chen, Ho, Lee, Wu, & Gau, 2017; Appendix S2). To cover the adulthood period, we used the Young Adult Self‐Report (Achenbach, 1997; 19–25 years) and the Adult Self‐Report (Achenbach & Rescorla, 2003; 33 years). The Achenbach scales have demonstrated good reliability and validity in Germany (Manfred, Klaus, Berner, Lehmkuhl, & Poustka, 1994; Walter, Remschmidt, & Deimel, 1994) and across different cultural contexts (Achenbach, 2019). The item numbers slightly differed across the assessment waves (Tables S5 and S6). Therefore, we applied proportion of maximum scaling to ensure the statistical equivalence of scores (Petersen et al., 2015).

#### Feature set

To predict psychopathology symptom trajectories, we used a wide range of biological and psychosocial measures. Biological measures included sex at birth (binary‐coded; 0 = male, 1 = female), polygenic scores (PGS) (e.g., aggression, anxiety), hormone levels (e.g., testosterone, cortisol), and obstetric risk (e.g., preterm birth). Given the profound relationship between childhood adversities and psychopathology (Baldwin et al., 2023; Daníelsdóttir et al., 2024), we included several adversity measures, including prenatal smoking, adverse family characteristics, income‐to‐need ratio, and stressful life events. Temperament, function level, and self‐perception measures were additionally included due to their link with psychopathology. A detailed description of each measure along with available psychometric properties can be found in Appendix S3. The feature set comprised 73 variables (Table S7), covering early life measures during childhood and adolescence (up to age 15). These developmental periods are pivotal for the emergence of psychopathology, offering a critical window to identify early risk factors and implement preventative strategies (McGrath et al., 2023).

### Data analysis

#### Trajectory modeling

To parse the heterogeneity in longitudinal symptom trajectories, we conducted growth mixture modeling implemented in MPlus (version 8.1). We defined a series of unconditional models ranging from one to five latent class solutions for internalizing and externalizing symptoms separately. Factors loadings corresponded to age at the measurement time. Missing data (4.5%) were handled by full information maximum likelihood procedure. The optimal number of latent classes was determined using multiple model fit indices. A good model fit is indicated by lower Bayesian and Akaike information criterion, higher entropy (>0.80), and significant likelihood ratio test (Vuong‐Lo‐Mendel‐Rubin and adjusted Lo‐Mendell‐Rubin).

#### Machine learning

Machine learning analyses were conducted in Python (3.12.4) using the scikit‐learn package. We used four models to predict class membership: Logistic regression, support vector machines, random forest, and XGBoost. IterativeImputer and the most frequent category were used to impute missing values for numerical and categorical (i.e., dummy‐coded) data, respectively. Continuous data were then scaled using MinMaxScaler. To prevent overfitting, we used a nested cross‐validation approach with 10 outer and five inner loops. We used RandomizedSearchCV for hyperparameter tuning. Class imbalance was handled using the parameter ‘class_weight’ (see Appendix S4 (Table S8) for a detailed description of the analysis pipeline). Macro and weighted F1 and area under the receiver‐operating characteristics curve (ROC AUC) [micro, macro, and class‐specific] were used to evaluate model performance. We computed Shapley Additive Explanations (SHAP) values to explain how much each feature contributed to a particular prediction.

### Supplemental analyses

We conducted a series of supplemental analyses to examine long‐term consequences of trajectory membership (adult functioning outcomes) and to explore whether symptom changes across development can be explained by key clinically relevant factors, including stress exposure, coping, resources, and treatment‐seeking. These analyses were intended to provide additional evidence for the validity and clinical relevance of the identified trajectories. Detailed descriptions of the measures and analytic procedures are provided in Appendix S5. Whereas the machine learning analyses were intentionally restricted to features measured in childhood and adolescence, the supplemental analyses incorporated additional variables assessed in adulthood.

#### Adult functioning outcomes

Trajectory groups were compared in terms of adulthood outcomes (e.g., education, income, substance use, and social functioning) using the measures collected at the last assessment wave (33 years).

#### Clinical and psychosocial correlates of symptom change

##### Treatment

We examined group differences in receiving treatment for psychological problems between T2 (age 2) and T11 (age 33).

### Stressful life events

Groups were compared in terms of total stress exposure (T1–T11) using a modified version of Munich Event List (Maier‐Diewald, Wittchen, Hecht, & Werner‐Eilert, 1983).

### Coping

To see if low symptoms were related to better coping skills, we compared the groups using coping skills ratings at T4 (age 8) and T5 (age 11) and the Stress Coping Styles Questionnaire (Janke & Erdmann, 2002) at T8 (age 22).

### Resources

Groups were compared in terms of social support and resources using standardized interviews at T4 (age 8) and T5 (age 11), and Resources Checklist (Dick, 2003) at T10 (age 25).

### Sensitivity analyses

To evaluate the robustness of the trajectory modeling, we repeated the growth mixture model analyses including all participants with at least three available assessments (*n* = 342), using the same model specifications as in the primary analyses. To assess the robustness and statistical significance of the machine learning predictions, we conducted permutation tests (100 iterations) on the best‐performing models to evaluate whether model performance exceeded chance level. Outcome labels were randomly shuffled and models were refitted using nested cross‐validation (5‐fold inner CV; 10‐fold outer CV) to generate null distributions for macro and class‐specific AUCs. Observed performance was compared against these null distributions to account for chance performance.

Although the features and outcome were distinct measures and did not overlap, the time points at which they were collected partially overlapped. Therefore, we conducted a sensitivity analysis using 45 features collected in childhood (up to age 8) to prove that models can capture meaningful patterns in data without overfitting. In addition, the symptom trajectory classes used as outcomes in the machine learning analyses were derived from psychopathology measures reported by parents and participants, while several predictors were also based on parent‐ and self‐reports. This overlap may introduce common informant bias, whereby associations are inflated because both predictors and outcomes originate from the same source. To evaluate whether our findings were influenced by such bias, we repeated the machine learning analyses using a separate feature set (43 features) based on objective and quasi‐objective measures (e.g., polygenic risk scores, hormone levels, observer‐rated risk scores, standardized tests).

Lastly, to handle missing data, we used *IterativeImputer*, a multivariate method that models missing values by repeatedly predicting each variable from the others until the estimates converge. To ensure that the choice of imputation did not affect our findings, we also evaluated model performance using alternative imputation strategies available in *sklearn.impute*, including mean imputation, median imputation, and K‐Nearest Neighbors imputation.

## Results

### Trajectory modeling

Mean trajectories of internalizing and externalizing symptoms across participants, as well as across subscales, are presented in Appendix S6 (Figures S1 and S2). We found a three‐class solution for both internalizing and externalizing problems: Increasing symptoms, decreasing symptoms, and low symptoms (Figure 1, see Tables S9 and S10 for model metrics). While trajectories were similar for internalizing and externalizing symptoms, peak times and rates of change differed (e.g., faster decline in externalizing symptoms). The majority of participants (~80%) were assigned to the low symptoms classes. Those in increasing symptoms classes had low childhood symptoms that rose in adolescence, peaking in young adulthood. Conversely, decreasing symptoms classes showed higher childhood symptoms that declined over time. Since both symptoms classes showed higher symptoms than the low symptoms classes at different periods (Tables S11–S14), we termed them risk trajectories. Overlap between symptom trajectories is shown in Figure S3.

**Figure 1:**
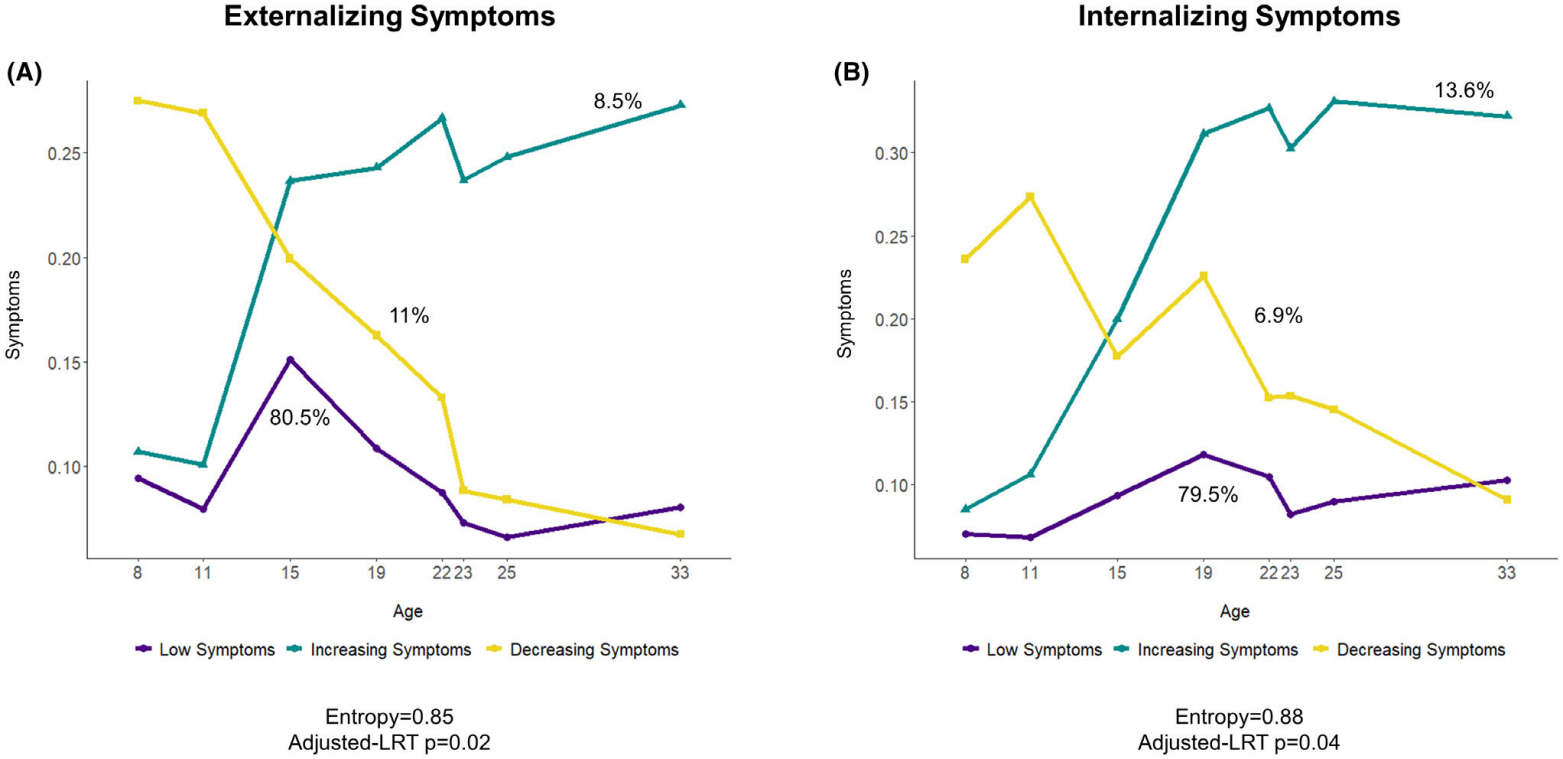
Identified class solution for externalizing (A) and internalizing (B) symptoms trajectories. Purple, green, and yellow colors represent low, increasing, and decreasing symptoms classes, respectively. Proportion of participants (%) belonging to the trajectory classes was shown for each symptom domain

### Machine learning

Using an extensive set of risk measures, we aimed to predict symptom classes. For externalizing symptoms trajectories, random forest achieved fair discriminatory power (Figure 2A), with a multiclass macro‐average AUC of 0.77 (SD = 0.07; 95% CI [0.72, 0.80]) and a multiclass micro‐average of 0.88 (SD = 0.04; 95% CI [0.86, 0.90]). F1‐based performance metrics showed a similar pattern (weighted F1 = 0.71, SD = 0.04, 95% CI [0.68, 0.73] and macro F1: AUC = 0.43, SD = 0.08, 95% CI [0.38, 0.47]). All trajectory classes were classified with acceptable discriminatory power (low symptoms: AUC = 0.74, SD = 0.10, 95% CI [0.69–0.80]; increasing symptoms: AUC = 0.77, SD = 0.07, 95% CI [0.73, 0.81]; decreasing symptoms: AUC = 0.78, SD = 0.13, 95% CI [0.70–0.86]).

**Figure 2:**
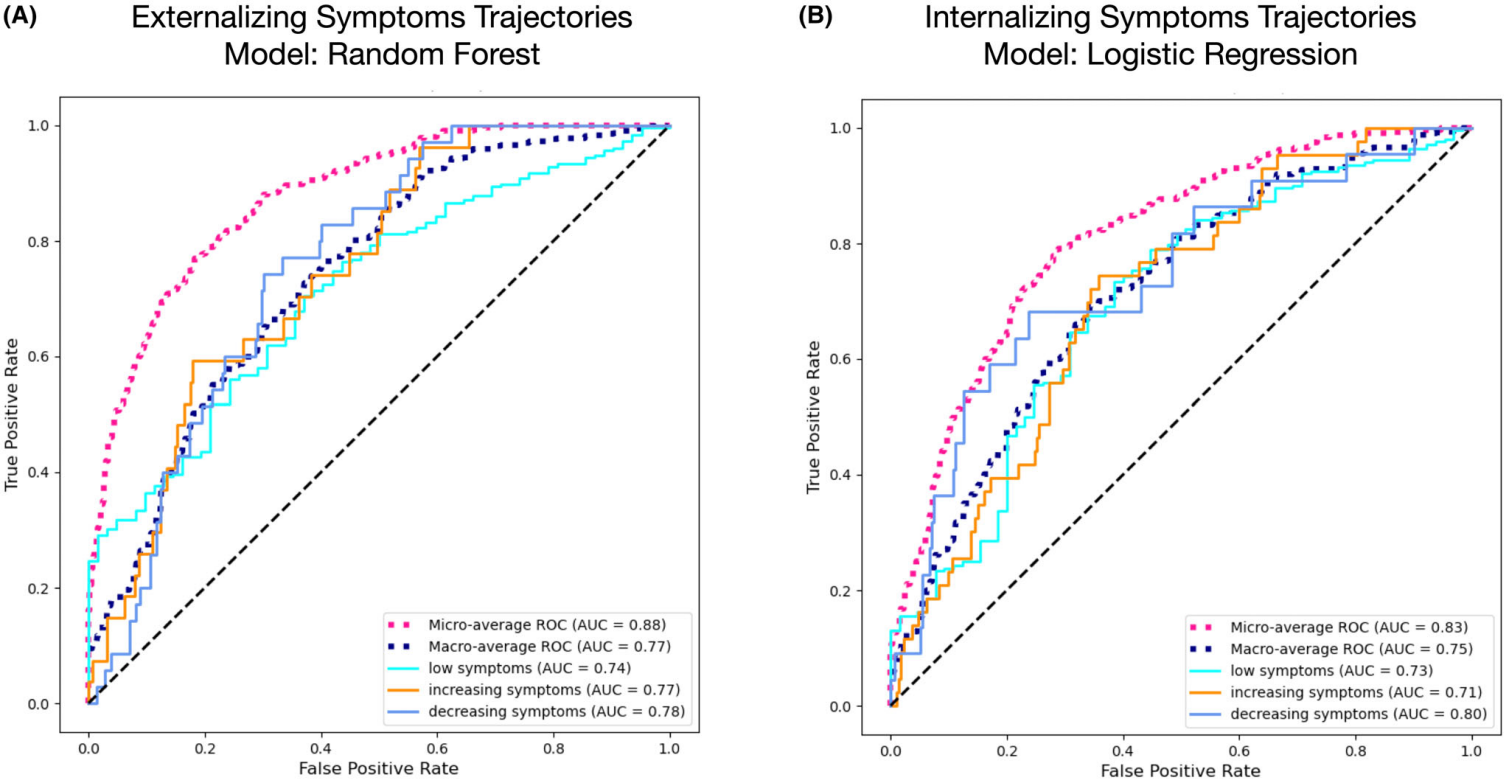
Multinominal classification performance for externalizing (A) and internalizing (B) symptoms trajectories. Discriminatory accuracy over all trajectories and for each individual class was shown for each symptom domain. AUC indicates area under curve. Diagonal line represents chance‐level performance

For internalizing symptoms trajectories, logistic regression achieved fair discriminatory power (Figure 2B), with a multiclass macro‐average AUC of 0.75 (SD = 0.09; 95% CI [0.69, 0.80]) and a multiclass micro‐average of 0.83 (SD = 0.06; 95% CI [0.80, 0.86]). F1‐based performance metrics showed a similar pattern (weighted F1 = 0.70, SD = 0.08, 95% CI [0.65, 0.75] and macro F1: AUC = 0.49, SD = 0.10, 95% CI [0.43, 0.55]). All trajectory classes were classified with mostly fair discriminatory power (low symptoms: AUC = 0.73, SD = 0.11, 95% CI [0.66, 0.80]; increasing symptoms: AUC = 0.71, SD = 0.13, 95% CI [0.64, 0.80]; decreasing symptoms: AUC = 0.80, SD = 0.17, 95% CI [0.69, 0.89]). Other machine learning models performed similarly (Appendix S7; Table S15).

### Trajectory specific features

The top 15 features for externalizing and internalizing symptoms trajectories are visualized in Figures 3 and 4, respectively (See Appendix S7 (Figures S4–S9) for the complete set of features with their contribution to model prediction).

**Figure 3:**
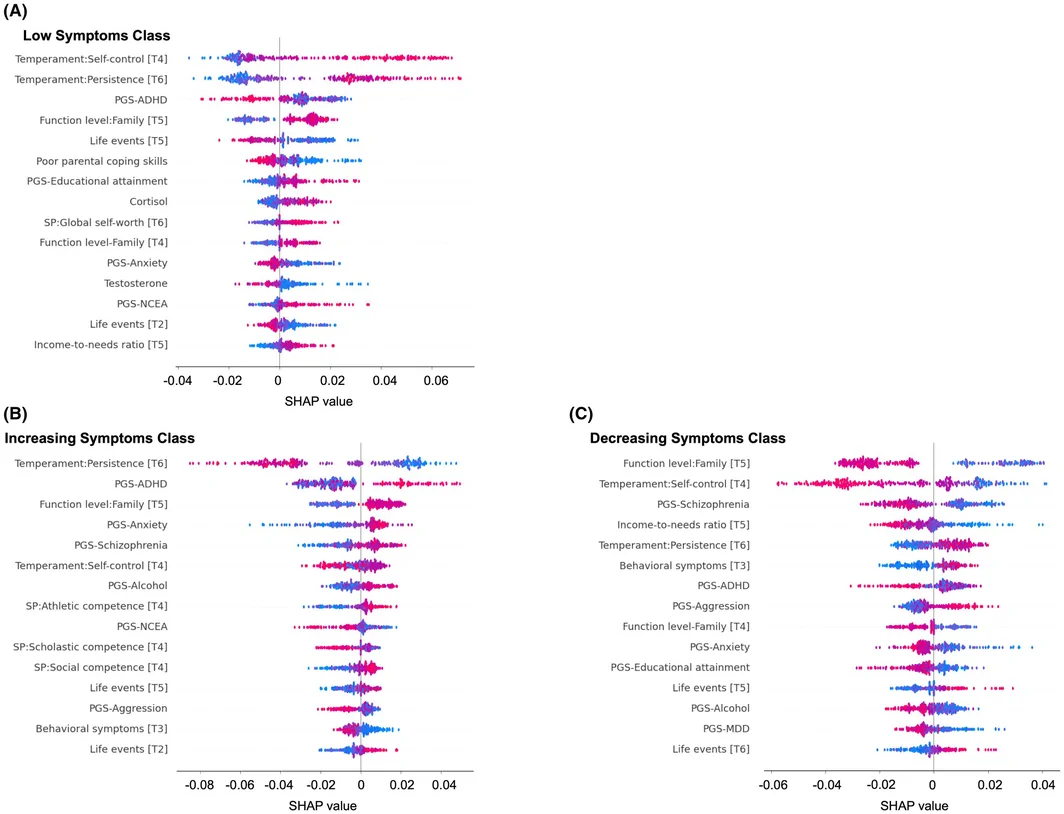
Top 15 features contributing to class‐specific model prediction for externalizing symptoms trajectories. Features on the *Y*‐axis are ranked by mean absolute SHAP value, with higher positions indicating greater overall contribution to the model. The *X*‐axis shows the distribution of SHAP values, where values to the right of zero indicate a positive contribution toward predicting the class and values to the left indicate a negative contribution. Color represents feature values, with blue indicating lower values and pink indicating higher values. Panels show low symptoms (A), increasing symptoms (B), and decreasing symptoms (C) trajectories. Measurement times were given for the same construct measured at different ages. ADHD, attention deficit and hyperactivity disorder; MDD, major depressive disorder; NCEA, non‐cognitive educational attainment; PGS, polygenic scores; SP, self‐perception. Measurement times: T1 = 3 months; T2 = 2 years; T3 = 4.5 years; T4 = 8 years; T5 = 11 years; T6 = 15 years

**Figure 4:**
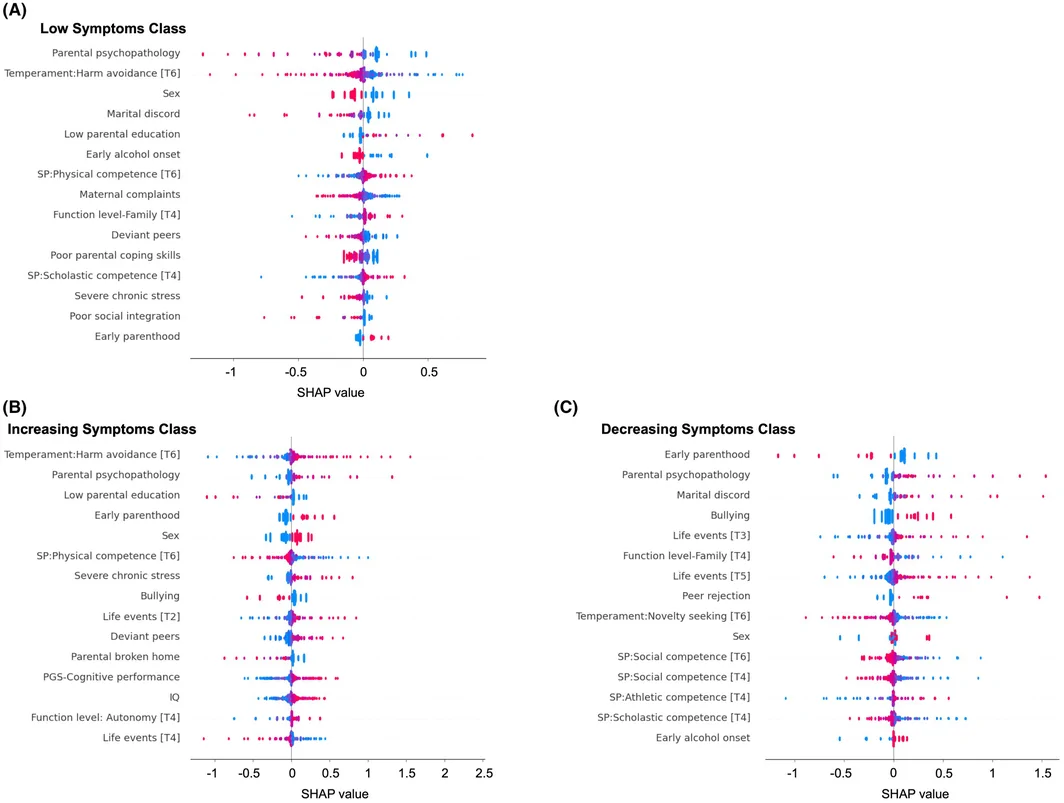
Top 15 features contributing to class‐specific model prediction for internalizing symptoms trajectories. Features on the *Y*‐axis are ranked by mean absolute SHAP value, with higher positions indicating greater overall contribution to the model. The *X*‐axis shows the distribution of SHAP values, where values to the right of zero indicate a positive contribution toward predicting the class and values to the left indicate a negative contribution. Color represents feature values, with blue indicating lower values and pink indicating higher values. Panels show low symptoms (A), increasing symptoms (B), and decreasing symptoms (C) trajectories. Measurement times were given for the same construct measured at different ages. PGS, polygenic scores; SP, self‐perception. Measurement times: T1 = 3 months; T2 = 2 years; T3 = 4.5 years; T4 = 8 years; T5 = 11 years; T6 = 15 years

#### Externalizing symptoms

Several protective factors, such as higher self‐control, higher persistence, lower PGS for anxiety and Attention Deficit and Hyperactivity Disorder (ADHD), protective family characteristics (better parental coping, higher income, higher family functioning), lower childhood stress, and lower testosterone levels were associated with a higher likelihood of being classified in the low symptoms trajectory.

Lower persistence, higher PGS for ADHD, anxiety, schizophrenia, and alcohol dependence, lower scholastic competence, and higher childhood stress were associated with higher likelihood of being classified in the increasing symptoms trajectory. In addition, the increasing trajectory was predicted by higher family functioning, higher self‐control, and higher social competence.

Several risk factors, such as lower family functioning, lower self‐control, lower family income, early behavioral problems, higher life stress, and higher PGS for aggression, were associated with a higher likelihood of being classified in the decreasing symptoms trajectory. Interestingly, the decreasing trajectory was predicted by higher persistence and lower PGS for several psychopathologies.

#### Internalizing symptoms

Several protective factors, such as positive family (e.g., parental harmony, social integration) and individual (e.g., lower harm avoidance, higher self‐competence) characteristics, and lower chronic stress, were associated with higher likelihood of being classified in the low symptoms trajectory.

Increasing symptoms trajectory was predicted by several risk factors, including higher harm avoidance, parental psychopathology, female sex, lower physical competence, higher life stress, and a higher number of deviant peers. Interestingly, increasing symptoms trajectory was also predicted by some protective factors, such as higher parental education, stable family environment, higher PGS for cognitive performance, higher IQ, and higher autonomy.

Several risk factors, such as parental psychopathology, marital discord, higher childhood stress, lower family and peer functioning (e.g., bullying, rejection), lower social and scholastic competence, and early alcohol onset, were associated with higher likelihood of being classified in the decreasing trajectory.

### Supplementary analyses

Results of the supplementary analyses are reported in Appendix S8.

#### Adult functioning

Risk trajectories were linked to adverse adulthood outcomes, including lower education, lower income, more hospitalization, a higher life‐time prevalence of mental health diagnosis, greater substance use (externalizing symptoms trajectories only), and poor social functioning (Tables S16–S19).

#### Treatment

Treatment timing aligned with peak symptoms periods. For both externalizing and internalizing problems, individuals in the decreasing symptoms trajectory were more likely to receive treatment in childhood, while those with increasing trajectories were treated more often at age 19 (Tables S20 and S21). Notably, only the increasing internalizing symptoms trajectory showed higher treatment rates in adulthood. This pattern was absent for externalizing symptoms.

#### Stressful life events

For both externalizing and internalizing problems, the risk trajectories had higher childhood stress compared with the low symptoms trajectories (Tables S22–S25). Only increasing symptoms trajectories had higher life stress during adulthood.

#### Coping

For both externalizing and internalizing problems, the decreasing symptoms trajectories had lower coping scores during childhood (Tables S26–S29), while the increasing symptoms trajectories used maladaptive coping strategies more often in adulthood (Tables S30–S33).

#### Resources

For both externalizing and internalizing problems, the decreasing symptoms trajectories had lower social support during childhood (Tables S34–S37), while the increasing symptoms trajectories had lower social and personal resources in adulthood (Tables S38–S41).

### Sensitivity analyses

Results of the sensitivity analyses are reported in Appendix S9. We replicated the three‐class solutions for both externalizing and internalizing symptoms in participants with at least three available assessments, with highly consistent growth trajectories and class distributions (Figure S10, Tables S42–S43). For both externalizing and internalizing symptom trajectories, observed model performance significantly exceeded chance‐level predictions. Macro AUCs were substantially higher than those derived from permutation‐based null distributions (externalizing: M = 0.77 vs. 0.49; internalizing: M = 0.75 vs. 0.51; both *p* < .01). Similarly, class‐specific AUCs for all trajectory groups were significantly greater than their corresponding null distributions, indicating robust and reliable predictive performance (Appendix S9).

The feature set covering the adolescence period provided better performance metrics, whereas the models using childhood features only showed slightly lower but above chance‐level performance (Figures S11–S13; Tables S44 and S45). Overall, model performance decreased slightly when analyses were restricted to objective and quasi‐objective features (Tables S46 and S47). For externalizing trajectories, predictive performance (macro AUC = 0.74) remained comparable to the primary analyses (macro AUC = 0.77). For internalizing trajectories, performance was reduced (macro AUC = 0.67 vs. 0.75). In addition, model performance was similar across the different imputation strategies (Tables S48 and S49).

## Discussion

Using longitudinal data from a birth cohort, we identified distinct developmental symptom trajectories through 25 years and demonstrated that these trajectories could be predicted with fair accuracy. Building on prior trajectory modeling research, this study uncovered several key biological and psychosocial contributors to risk (e.g., higher PGS for psychiatric disorders, childhood adversities, social dysfunction). Notably, risk trajectories were also predicted by some protective features (e.g., higher family functioning, higher cognitive capacity), which might delay but not necessarily prevent symptom onsets. Moreover, childhood‐onset problems were primarily characterized by modifiable environmental factors, highlighting the potential of early interventions to mitigate long‐term adverse outcomes. Finally, ongoing stress, poor coping skills and lack of social resources were related to poorer prognosis, which need to be addressed by comprehensive intervention strategies.

We identified similar trajectories for externalizing and internalizing symptoms, with slight differences in peaks and growth patterns. Consistent with previous literature (Fernandez Castelao & Kröner‐Herwig, 2014; Kemmis‐Riggs et al., 2020; Klinge et al., 2023; Nivard et al., 2017; Weeks et al., 2014), most participants had low symptoms throughout development, representing largely normative emotional and behavioral functioning with modest fluctuations during sensitive periods such as adolescence. In these low symptom groups, both symptom types peaked around adolescence (ages 15–19), consistent with findings from epidemiological studies (McGrath et al., 2023) and trajectory modeling research (Bevilacqua et al., 2021; Petersen et al., 2015), highlighting adolescence as a sensitive period for mental health. A small proportion showed increasing symptoms, capturing escalating symptom levels over development and is consistent with a latent vulnerability framework (i.e., underlying psychological risk manifests later in response to increasing developmental or environmental demands) (McCrory & Viding, 2015). Another small proportion exhibited elevated symptoms early in life followed by attenuation over time (decreasing symptoms), suggesting developmental adaptation or recovery, potentially related to maturation of regulatory systems or improvements in environmental context. We did not identify a chronically high symptom group, possibly due to small sample size or the 25‐year measurement period.

Higher early life stress was a significant predictor of both risk trajectories for externalizing symptoms. The increasing trajectory was associated with lower persistence traits and high genetic liability for several psychopathologies despite intact family functioning and social competence. By contrast, the decreasing (childhood‐limited) trajectory was associated with higher PGS for aggression, yet predominantly predicted by psychosocial risk factors (e.g., lower family functioning and income). Notably, PGS proved to be valuable predictors of risk trajectories for externalizing symptoms, with their impact being particularly pronounced for adolescent‐onset problems. These findings align with the previous research reporting higher PGS among individuals with heightened externalizing symptoms trajectories (Speyer et al., 2022).

Elevated life stress and parental psychopathology were common in both risk trajectories for internalizing symptoms. The increasing (late‐onset) trajectory was associated with a higher harm avoidance, female sex, and lower physical competence during adolescence. Interestingly, high intellectual capacity (e.g., higher IQ, PGS for cognitive performance), greater autonomy, and higher parental education also predicted group membership. While this might seem paradoxical, high intellectual capacity and social position could foster heightened expectations and perfectionist tendencies (Lyman & Luthar, 2014), which in turn might increase the susceptibility to internalizing problems (Fredrick, Demaray, & Jenkins, 2017). Its protective effects may be limited and likely interact with other risk factors. The decreasing (childhood‐onset) trajectory was characterized by lower family functioning, impaired social competence, and high peer‐related adversity (e.g., rejection, bullying). Consistent with prior research (Korhonen et al., 2014; Rappaport et al., 2021), these findings highlight the link between internalizing symptoms and social dysfunctions.

The risk trajectories were associated with not only by adverse factors but also with protective variables (e.g., higher family functioning, intellectual capacity), particularly in the case of increasing symptoms trajectories. This pattern might reflect delayed manifestation of symptoms despite elevated early risk. However, while protective factors might postpone onset of problems, they do not necessarily prevent them. By contrast, childhood‐onset problems were primarily related to adverse environmental factors and showed no strong association with protective factors. Despite their early vulnerability, this group exhibited alleviation in their symptoms over time, potentially reflecting natural recovery through developmental (e.g., gaining independence) or external (e.g., normalization of social environment) processes.

Both risk trajectories for externalizing and internalizing symptoms were linked to long‐term negative outcomes in adulthood (e.g., lower education, increased hospitalizations, living alone), highlighting the need for effective intervention and prevention strategies. Despite increased treatment‐seeking with higher symptoms, treatment rates remained low (<50%), consistent with a prior meta‐analysis (Wang et al., 2023). Childhood‐onset trajectories were largely characterized by family dysfunction, with internalizing symptoms also linked to poor peer relationships and low social competence, suggesting that interventions focusing on social skills and caregiver involvement may be beneficial (Dietz, 2020). In contrast, adolescent‐onset problems often persisted into adulthood. Chronic externalizing symptoms may be explained by genetic liability and low treatment‐seeking, while the mechanisms behind persistent internalizing symptoms remain less clear. While adulthood stress likely play a key role, bidirectional relationships between stress and psychopathology may also perpetuate symptoms (e.g., stress sensitization) (Jenness, Peverill, King, Hankin, & McLaughlin, 2019). Strengthening coping strategies and social skills could potentially alleviate symptoms and disrupt the cycle of chronicity.

Our machine learning models successfully identified meaningful patterns and provided a useful framework for identifying factors contributing to heterogeneous symptom trajectories. However, several limitations should be acknowledged. First, imbalanced class distribution and small sample size limits the performance and generalizability of machine learning models. Although deep phenotyping and careful methodological choices (e.g., nested cross‐validation, permutation testing) may have partially mitigated these constraints, classification performance was modest and remains insufficient for individual‐level clinical application. The absence of an independent external validation sample further limits conclusions about robustness and generalizability. Larger samples and external validation are needed for more robust findings. Second, we used raw scores to model symptom changes across development (Petersen et al., 2015). Although high scores indicate risk, they do not reflect diagnosis. Third, our analysis did not include preschool period, which can be an important period for the onset of some psychopathologies (Bufferd, Dougherty, Carlson, Rose, & Klein, 2012). In addition, although we repeatedly collected data from childhood to adulthood at representative ages, relatively long intervals between some assessment waves (range: 1–8 years, mean = 3.5 years) may have led to information loss in trajectory modeling and limited our ability to capture shorter‐term fluctuations and more complex developmental patterns. Fourth, although SHAP values provide valuable insights into model behavior and enhance interpretability, they are dependent on the underlying model and data reflect associations rather than causal relationships. Additionally, they do not indicate independent importance, as variance can be shared among correlated or interacting predictors. Therefore, the identified features should be interpreted as descriptive contributions within this specific model, rather than as universal or causal determinants. Fifth, while the majority of measures used in this study were well‐established and psychometrically validated, some predictors consisted of single‐item indicators or measures lacking full psychometric evaluation. Findings involving measures with limited psychometric information should therefore be interpreted with caution. Lastly, most of the participants are Caucasian, which limits the generalizability of these findings to individuals from other racial backgrounds.

## Conclusions

Our findings suggest that psychopathology symptom trajectories evolve dynamically across development and can be predicted by a combination of genetic and psychosocial factors. The identified risk factors offer valuable insights for early identification and the design of targeted prevention strategies, potentially mitigating the long‐term consequences of mental health challenges. Future research should extend these findings by incorporating larger samples and employing more frequent symptom assessments over an extended time period, which would increase statistical power and improve sensitivity to short‐term changes.

## Ethical considerations

The study was approved by the Medical Ethics Committee II of Heidelberg University, Germany (application number: 2015‐612N‐MA; approval date: 10.11.2015), as part of the project ‘Exploring the long‐term impact of early life adversity on the social brain’. Written informed consent was obtained from all participants prior to participation. For assessment waves conducted while participants were minors, written informed consent was obtained from both the participants and their legal guardians. All procedures were conducted in accordance with the Declaration of Helsinki.

**Key points**

**What's known?**

- Psychopathology symptom trajectories vary considerably across development and are associated with a range of psychosocial factors.

**What's new?**

- Using well‐phenotyped data from participants followed over 30 years, we demonstrated that psychopathology symptom trajectories vary across development in terms of onset and progression and are shaped by both genetic and psychosocial factors.
- Machine learning models achieved modest discriminatory power in predicting class membership. Key features for prediction included life stress, polygenic scores, adverse family characteristics, temperament, social functioning, and self‐perception.

**What's relevant?**

- The ability to predict trajectory membership highlights opportunities for early identification and targeted prevention strategies.

## Supporting information


**Appendix S1.** Sample characteristics.
**Table S1.** Participation rate across the waves.
**Table S2.** Participants number with available psychopathology data.
**Table S3.** Overall missing data pattern.
**Table S4.** Attrition bias analysis.
**Appendix S2.** Psychopathology measures.
**Table S5.** Internalizing symptoms.
**Table S6.** Externalizing symptoms.
**Appendix S3.** Features used in machine learning analysis.
**Table S7.** Feature list including measures collected up to age 15.
**Appendix S4.** Machine learning pipeline.
**Table S8.** Hyperparameter settings.
**Appendix S5.** Measures for supplemental analyses.
**Appendix S6.** Extended results for trajectory modeling.
**Figure S1.** Mean trajectories for internalizing and externalizing symptoms across development.
**Figure S2.** Mean symptom trajectories for the subscales.
**Table S9.** Model fit indices of latent growth mixture models for internalizing symptoms.
**Table S10.** Model fit indices of latent growth mixture models for externalizing symptoms.
**Figure S3.** Overlap between symptom trajectories.
**Table S11.** Comparisons of externalizing symptoms trajectories in terms of externalizing symptoms.
**Table S12.** Post‐hoc tests for group comparisons: externalizing symptoms trajectories.
**Table S13.** Group comparisons of internalizing symptoms trajectories in terms of internalizing symptoms.
**Table S14.** Post‐hoc tests for group comparisons: internalizing symptoms.
**Appendix S7.** Extended results for machine learning analysis.
**Table S15.** Model performance metrics for the prediction of symptom trajectories.
**Figure S4.** Full list of 73 features contributing to model prediction of low symptoms trajectory class for externalizing symptoms.
**Figure S5.** Full list of 73 features contributing to model prediction of increasing symptoms trajectory class for externalizing symptoms.
**Figure S6.** Full list of 73 features contributing to model prediction of decreasing symptoms trajectory class for externalizing symptoms.
**Figure S7.** Full list of 73 features contributing to model prediction of low symptoms trajectory class for internalizing symptoms.
**Figure S8.** Full list of 73 features contributing to model prediction of increasing symptoms trajectory class for internalizing symptoms.
**Figure S9.** Full list of 73 features contributing to model prediction of decreasing symptoms trajectory class for internalizing symptoms.
**Appendix S8.** Results for supplementary analyses.
**Table S16.** Comparisons of externalizing symptom trajectories in terms of adult functioning.
**Table S17.** Post‐hoc tests for group comparisons: externalizing symptom trajectories.
**Table S18.** Comparisons of internalizing symptom trajectories in terms of adult functioning.
**Table S19.** Post‐hoc tests for group comparisons: internalizing symptom trajectories.
**Table S20.** Treatment use for psychological problems: externalizing symptom trajectories.
**Table S21.** Treatment use for psychological problems: internalizing symptom trajectories.
**Table S22.** Comparisons of externalizing symptoms trajectories in terms of stress life events.
**Table S23.** Post‐hoc tests for group comparisons: externalizing symptom trajectories.
**Table S24.** Comparisons of internalizing symptom trajectories in terms of stress life events.
**Table S25.** Post‐hoc tests for group comparisons: internalizing symptom trajectories.
**Table S26.** Comparisons of externalizing symptom trajectories in terms of coping ratings.
**Table S27.** Post‐hoc group comparisons: externalizing symptom trajectories.
**Table S28.** Comparisons of internalizing symptom trajectories in terms of coping ratings.
**Table S29.** Post‐hoc tests for group comparisons: internalizing symptom trajectories.
**Table S30.** Comparisons of externalizing symptom trajectories in terms of stress coping styles in adulthood (T8).
**Table S31.** Post‐hoc tests for group comparisons: externalizing symptom trajectories.
**Table S32.** Comparisons of internalizing symptom trajectories in terms of stress coping styles in adulthood (T8).
**Table S33.** Post‐hoc tests for group comparisons: internalizing symptom trajectories.
**Table S34.** Comparisons of externalizing symptom trajectories in terms of social support.
**Table S35.** Post‐hoc tests for group comparisons: externalizing symptom trajectories.
**Table S36.** Comparisons of internalizing symptom trajectories in terms of social support.
**Table S37.** Post‐hoc tests for group comparisons: internalizing symptom trajectories.
**Table S38.** Comparisons of externalizing symptom trajectories in terms of resources in young adulthood.
**Table S39.** Post‐hoc tests for group comparisons: externalizing symptom trajectories.
**Table S40.** Comparisons of internalizing symptom trajectories in terms of resources in young adulthood.
**Table S41.** Post‐hoc tests for group comparisons: internalizing symptom trajectories.
**Appendix S9.** Sensitivity analyses.
**Figure S10.** Identified class solution for externalizing (A) and internalizing (B) symptoms trajectories. Purple, green, and yellow colors represent low, increasing, and decreasing symptoms classes, respectively.
**Table S42.** Model fit indices of latent growth mixture models for externalizing symptoms (*n* = 342).
**Table S43.** Model fit indices of latent growth mixture models for internalizing symptoms (*n* = 342).
**Table S44.** Feature List including measures collected up to age 8.
**Figure S11.** Multinominal classification performance for externalizing (A) and internalizing (B) symptom trajectories.
**Figure S12.** Top 15 features contributing to class‐specific model prediction for externalizing symptoms trajectories. Features on the *Y*‐axis are ranked by mean absolute SHAP value, with higher positions indicating greater overall contribution to the model. The *X*‐axis shows the distribution of SHAP values, where values to the right of zero indicate a positive contribution toward predicting the class and values to the left indicate a negative contribution. Color represents feature values, with blue indicating lower values and pink indicating higher values. Panels show low symptoms (A), increasing symptoms (B), and decreasing symptoms (C) trajectories. ADHD, attention deficit and hyperactivity disorder; MDD, major depressive disorder.
**Figure S13.** Top 15 features contributing to class‐specific model prediction for internalizing symptoms trajectories. Features on the *Y*‐axis are ranked by mean absolute SHAP value, with higher positions indicating greater overall contribution to the model. The *X*‐axis shows the distribution of SHAP values, where values to the right of zero indicate a positive contribution toward predicting the class and values to the left indicate a negative contribution. Color represents feature values, with blue indicating lower values and pink indicating higher values. Panels show low symptoms (A), increasing symptoms (B), and decreasing symptoms (C) trajectories. ADHD, attention deficit and hyperactivity disorder; MDD, major depressive disorder; NCEA, non‐cognitive educational attainment; PGS, polygenic scores. Measurement times: T1 = 3 months; T2 = 2 years; T3 = 4.5 years; T4 = 8 years.
**Table S45.** Feature list based on information source.
**Table S46.** Model performance metrics for externalizing symptom trajectories across feature sets.
**Table S47.** Model performance metrics for internalizing symptom trajectories across feature sets.
**Table S48.** Model performance metrics across different imputation techniques: externalizing symptoms trajectories.
**Table S49.** Model performance metrics across different imputation techniques: internalizing symptoms trajectories.

## Data Availability

The analysis code used to generate the published findings is available in the first author's GitHub repository (https://github.com/sedasacu/symptom‐trajectories‐ml/). Due to ethical constraints and participant consent restrictions in the Mannheim Study of Children at Risk, the raw participant data cannot be shared publicly or with third parties.
