# Supplementary material for: Predicting psychopathology symptom trajectories using machine learning: a 33‐year prospective study
Source: J Child Psychol Psychiatry. 2026 Jun 15;67(11):1855–67. doi: 10.1111/jcpp.70180 (PMC13647092; doi:10.1111/jcpp.70180)
Supplement: Supplementary file 1 [file JCPP-67-1855-s001.docx]

**Predicting psychopathology symptom trajectories using machine learning:**

**A 33-year prospective study**

**Supporting Information**

# Table of Contents

Appendix S1. Sample Characteristics	3

Appendix S2. Psychopathology Measures	7

Parent versus Self-Report	8

Appendix S3. Features Used in Machine Learning Analysis	9

Biological Measures	9

Psychosocial Risk Measures	11

Other Measures	15

Appendix S4. Machine Learning Pipeline	23

Appendix S5. Measures for Supplemental Analyses	25

Adult Functioning	25

Clinical and Psychosocial Correlates of Symptom Change	26

Appendix S6. Extended Results for Trajectory Modeling	29

Group Mean	29

Trajectory Modeling with Latent Growth Mixture Modeling	30

Overlap between Symptom Trajectories	32

Group Comparisons using Symptom Scores	32

Appendix S7. Extended Results for Machine Learning Analysis	35

Model Performance	35

Trajectory Specific Features	37

Appendix S8. Results for Supplementary Analyses	44

Adulthood Outcomes	44

Clinical and Psychosocial Correlates of Symptom Change	49

Appendix S9. Sensitivity Analyses	65

Trajectory Modeling with Full Available Data	65

Permutation Tests	67

Model Predictions with Childhood Features Only	68

Common Informant Bias	74

The Impact of Imputation Strategies	77

References	80

# Appendix S1. Sample Characteristics

The initial sample included 384 infants recruited from two obstetric and six children´s hospitals in the Rhine-Neckar region of Germany between 1986 and 1988. Participants were followed from their birth up to around the age of 33 years (age range: 31.7- 34.5 years) across 11 assessment waves (Table S1). At the last assessment wave (T11), 256 participants (67%) agreed to participate in the study and completed several psychological measurements.

**Table S1.** Participation rate across the waves.

|  | **T1**  (3 m) | **T2**  (2 y) | **T3**  (4.5 y) | **T4**  (8 y) | **T5**  (11 y) | **T6**  (15 y) | **T7**  (19 y) | **T8**  (22 y) | **T9**  (23 y) | **T10**  (25 y) | **T11**  (33 y) |
| --- | --- | --- | --- | --- | --- | --- | --- | --- | --- | --- | --- |
| **N**  (%) | 384 | 376  (98%) | 373  (97%) | 370  (96%) | 364  (95%) | 338  (88%) | 327  (85%) | 315  (82%) | 312  (81%) | 309  (80%) | 256  (67%) |

Abbreviations: m, months; y, years.

Psychopathology symptoms were measured from eight years (T4) on across eight assessment waves (Table S2-S3). To reliably model the psychopathology symptom trajectories, we included the participants who had available data from at least five assessment waves (n=317, 171 females).

**Table S2.** Participants number with available psychopathology data.

|  | **T4**  (8 y) | **T5**  (11 y) | **T6**  (15 y) | **T7**  (19 y) | **T8**  (22 y) | **T9**  (23 y) | **T10**  (25 y) | **T11**  (33 y) |
| --- | --- | --- | --- | --- | --- | --- | --- | --- |
| **N**  (%) | 344  (90%) | 352  (92%) | 337  (88%) | 326  (85%) | 315  (82%) | 312  (81%) | 309  (80%) | 254  (66%) |

**Table S3.** Overall Missing Data Pattern.

| **Missing** | **N** | **Cumulative N (%)** |
| --- | --- | --- |
| None | - | 224 (58%) |
| One time point | 76 | 300 (78%) |
| Two time points | 14 | 314 (82%) |
| Three time points | 3 | 317 (83%) |
| Four time points and more | 53 | 370 (96%) |
| All time points (no data) | 14 | 384 (100%) |

Among 317 participants, missing data pattern across waves was completely at random (Little’s MCAR test; Chi-square=92.57, p=0.08).

Mannheim Study of Children at Risk was specifically designed to investigate long-term impact of biological and psychosocial risk factors on development. We, therefore, compared the included (n=317) and excluded participants (n=67) in terms of risk factors covering early life (i.e., from prenatal period to late childhood), such as obstetric risk (e.g., birth complications, low birth weight), childhood adversities (e.g., family adversity, prenatal smoking), and life stress. Chi-square test is conducted to see if there are group differences in terms of sex and prenatal maternal smoking. Two-sample t-test was performed to identify group differences in continuous variables. Mann-Whitney U test was performed, when assumptions for the parametric test were not met. Groups differed in terms of prenatal maternal smoking, peer rejection, and family adversity. Excluded participants had higher proportion of mothers smoking during pregnancy, higher proportion of peer rejection at age 11, and higher family adversity in the first three assessment waves compared to the included participants (Table S4). Groups did not differ in terms of other childhood adversities and life stress.

**Table S4.** Attrition Bias Analysis.

| **Measurements ^a^** | **Time** | **Included (n=317)** | **Excluded (n=67)** | **Test-statistics** | **df** | **p** |
| --- | --- | --- | --- | --- | --- | --- |
| Sex, N, male/female | - | 146/171 | 39/28 | 3.27 | 1 | 0.07 |
| Prenatal maternal smoking, N,  no/moderate/heavy smoker | T1 | 238/31/48 | 39/12/16 | 7.98 | 2 | **0.018*** |
| Prenatal maternal stress | T1 | 3.00 (2.07) | 3.01 (1.96) | 0.05 | 382 | 0.96 |
| Maternal psychological complaints, M (SD)b | T1 | 46.26 (10.05) | 45.86 (11.27) | -0.29 | 382 | 0.77 |
| Maternal stimulation ^b^, M (SD) | T1 | -0.02 (2.51) | 0.09 (2.58) | 0.31 | 368 | 0.76 |
| Income-to-need ratio ^c^, M (SD) | T1 | 0.17 (0.22) | 0.13 (0.19) | -1.17 | 382 | 0.24 |
| Income-to-need ratio ^c^, M (SD) | T5 | 0.20 (0.17) | 0.20 (0.18) | 0.09 | 358 | 0.93 |
| Peer rejection, N, no/yes | T5 | 281/32 | 29/19 | 29.57 | 1 | **0.000***** |
| Bullying, N, no/yes | T5 | 225/65 | 22/10 | 0.95 | 1 | 0.33 |
| Family adversity ^d^, Median (IQR) | T1 | 1 (3) | 2 (3) | 8391 | 384 | **0.006**** |
| Family adversity ^d^, Median (IQR) | T2 | 1 (3) | 2 (3) | 7408 | 377 | **0.006**** |
| Family adversity ^d^, Median (IQR) | T3 | 1 (3) | 2 (3) | 7411 | 373 | **0.044*** |
| Family adversity, M (SD) | T4 | 1.77 (1.70) | 2.12 (1.77) | 1.35 | 367 | 0.18 |
| Family adversity, M (SD) | T5 | 1.48 (1.57) | 1.89 (1.78) | 1.61 | 358 | 0.11 |
| Stressful life events, M (SD) | T1 | 3.84 (2.37) | 4.07 (2.08) | 0.74 | 382 | 0.46 |
| Stressful life events, M (SD) | T2 | 5.41 (3.03) | 5.95 (3.47) | 1.21 | 371 | 0.23 |
| Stressful life events, M (SD) | T3 | 6.96 (3.49) | 7.05 (3.36) | 0.18 | 371 | 0.86 |

| Stressful life events, M (SD) | T4 | 6.69 (3.60) | 6.70 (4.36) | 0.01 | 370 | 0.99 |
| --- | --- | --- | --- | --- | --- | --- |
| Stressful life events, M (SD) | T5 | 5.44 (3.41) | 4.81 (2.94) | -1.21 | 362 | 0.23 |

^a^ See Supplementary Material S2 for the detailed description of measurements^b^ Z-transformed scores	^c^ Log-scaled scores ^d^ Mann Whitney U Test	* p<0.05	* p <0.01

# Appendix S2. Psychopathology Measures

Internalizing symptom scores were based on anxious/depressed, withdrawn, and somatic complaints scales, while externalizing symptoms scores were based on aggressive behavior, rule-breaking, and intrusive scales. Table S5 & S6 show the item numbers for each subscale for internalizing and externalizing symptoms scores, respectively.

**Table S5.** Internalizing Symptoms.

|  | Anxious-Depressed | Withdrawn | Somatic Complaints | Total |
| --- | --- | --- | --- | --- |
| T4 | 14 | 9 | 9 | **31** |
| T5 | 14 | 9 | 9 | **31** |
| T6 | 16 | 7 | 9 | **31** |
| T7 | **17** | **7** | 12 | **36** |
| T8 | **17** | **7** | 12 | **36** |
| T9 | 17 | 7 | 12 | **36** |
| T10 | 17 | 7 | 12 | **36** |
| T11 | 18 | 9 | 12 | **39** |

**Table S6.** Externalizing Symptoms.

|  | Rule-Breaking Behavior | Aggressive Behavior | Intrusive | Total |
| --- | --- | --- | --- | --- |
| T4 | 13 | 20 | NA | **33** |
| T5 | 13 | 20 | NA | **33** |
| T6 | 11 | 19 | NA | **30** |
| T7 | 9 | 12 | _7_a | **28** |
| T8 | 9 | 12 | _7_a | **28** |
| T9 | 9 | 12 | _7_a | **28** |
| T10 | 9 | 12 | _7_a | **28** |
| T11 | 14 | 15 | _6_a | **35** |

^a^ The items were previously gathered under aggressive behavior scale.

# Parent versus Self-Report

At age 15, both parents and youths reported youths’ psychological symptoms using the Child Behavior Checklist(1) and Youth Self-Report(1), respectively. The scores were not normally distributed. Therefore, to estimate agreement between parent- and self-reported psychopathology symptoms, we performed a Spearmen Correlation Rank Test, which revealed moderate correlations between parent- and self-reported symptoms for internalizing (r_s_=0.38, p=0.000) and externalizing (r_s_=0.40, p=0.000) problems. These findings are consistent with previous research showing moderate agreement between parent- and self-reported internalizing and externalizing symptoms using the same psychopathology measures (2,3).

We also conducted a paired t-test to determine whether there were significant differences between parent- and self-reported symptoms. Interestingly, youth reported higher internalizing (Self-Report: Med=6, IQR=7; Parent-Report: Med=4, IQR=5; t(310)=4.09, p=0.000) and higher externalizing symptoms (Self-Report: Med=9, IQR=7; Parent-Report: Med=4, IQR=7; t(310)=9.31, p=0.000) compared to their parents.

Parental psychopathology was assessed using diagnostic interviews between T1 (3 months) and T5 (age 11). Since the previous literature has shown that parental psychopathology might influence parent-reports (4), we conducted a sensitivity analysis to identify the impact of parental psychopathology on symptom ratings. For this purpose, we first calculated a difference score (Self-report score – parent-report score) for internalizing and externalizing symptoms for each individual. Then, a non-parametric two-sample t-test (Mann-Whitney U Test) was performed to examine potential group differences. We found no significant group difference in internalizing symptom scores between youths with or without parental psychopathology. However, youths without parental psychopathology (Med=5, IQR=7) rated their externalizing symptoms higher than those with parental psychopathology (Med=3, IQR=8.75) (z=2.18, p=0.029).

# Appendix S3. Features Used in Machine Learning Analysis

Feature sets included a wide range of variables in biological, psychological and social domains.

# Biological Measures

**Sex (T1).** Sex referred to being male or female at birth and was coded as a binary variable (0 = male, 1 = female).

**Obstetric Risk (T1).** Obstetric risk scores were calculated according to the degree of obstetric complications based on medical reports (5). The score range is 0-4 (0= no risk, 1-2=moderate risk, 3-4= high risk). 108 infants (34.07%) were born full-term, had normal birth weights and had no medical complications. 209 infants had moderate to high biological risk (moderate = 56.47%, e.g., preterm birth, preterm labor; high = 9.46%, e.g., birth weight < 1500 g, neonatal complications).

**Polygenic scores (T6).** DNA was extracted from whole blood or saliva of 306 participants of the initial sample. Genome-wide genotyping was performed using Global Screening Array 24 version 2 (Illumina, Inc., San Diego, CA, USA) at the Life & Brain facilities, Bonn, Germany

(6). Quality control and filtering was performed using PLINK v1.90b6.7 (7), removing participants with >.02 missingness, heterozygosity rate >|.20|, and sex mismatch. SNPs with a minor allele frequency of <.01, deviating from Hardy-Weinberg equilibrium (HWE) with a p-value of <10-6 and missing data >.02 were removed. Relatedness and population structure were filtered based on a SNP set filtered for high quality (HWE P>.02, MAF>.20, missingness = 0), and LD pruning (r²=.1). If subjects were cryptically related (pi hat>.10), one subject was excluded at random. Control for population stratification was performed by generating principal components and outliers, defined as deviating more than four SD on one of the first 20 principal components were excluded. After quality control and filtering, the final sample included 299 individuals and 482,981 SNPs.

For the current study, we included 10 polygenic scores that were calculated based on the genome-wide association data: attention deficit and hyperactivity disorder (8), aggression (9), alcohol dependence (10), anxiety (11), cognitive performance (12), educational attainment (12), intelligent quotient (13), non-cognitive educational attainment (14), major depressive disorder (including data from research-consented participants of 23andMe, Inc.) (15), and schizophrenia

(16). Polygenic scores were calculated with PRSice version 2.2.6 (17) after clumping SNPS (linkage disequilibrium *r*² < 0.1 250 kb sliding window) for multiple *p*-value thresholds. For the analysis in the present study, we selected the p-value of 0.05. We used residualized polygenic scores as features in the machine learning analyses (18). The first 10 principal components were regressed out using resid() function in R.

**Hormone Levels (T6).** All participants were asked to abstain from alcohol, smoking, sport activities and a heavy lunch (19). Blood sample (35 ml) was taken between five and six p.m. The samples were collected in polypropylene tubes containing citric acid and centrifuged at for 10 min. Plasma was stored at –80 °C until analysis. Testosterone (T), Dihydrotestosterone (DHT), and cortisol were measured with in-house RIAs after extraction and chromatographic separation. Briefly, extracts of 500 μl plasma were chromatographed on celite mini-columns (750 mg) with propylene glycol as a stationary phase. The DHT-fraction was eluted with 8% ethyl acetate in i-octane, followed by T (20% ethyl acetate in i-octane) and quantified with specific RIAs. RIA results were corrected with individually (for each sample) determined recovery (for DHT 60% and T 78%). The sensitivity, intraassay variation and interassay variation were 1 pg, less than 7% and less than 18% for DHT, and 1 pg, less than 5% and less than 14% for T, respectively (20). Intra-assay variation for plasma cortisol was less than 5%, inter-assay variation was less than 7%. The limit of detection was 5 pg (19).

# Psychosocial Risk Measures

**Prenatal Maternal Smoking (T1).** We measured maternal smoking during pregnancy by a standardized interview conducted with mothers at the 3-month assessment (21). Mothers were asked about their daily cigarette consumption (none, moderate smoking= up to 5 per day, heavy smoking= more than 5 per day). Of the mothers of participants, 238 (75.08%) were nonsmokers, 31 (9.78%) reported smoking 1 to 5 cigarettes per day, and 48 (15.14%) reported smoking more than five cigarettes per day.

**Prenatal Maternal Stress (T1).** Prenatal maternal stress was measured at the first assessment wave (age of 3 months) via a standardized parent interview conducted by trained interviewers

(22). Mothers answered 11 questions covering negative experiences (e.g., ‘Did you have mood swings/a depressed mode’) and negatively coded positive experiences (e.g., ‘Did you look forward to having a baby’) during pregnancy. Additionally, mothers were asked about the timing of these experiences (the first and the second/third trimesters). Internal consistency was acceptable (Cronbach’s alpha= 0.72 for the first trimester and 0.65 for the second and third trimester) (22). Here, we specifically used the prenatal maternal stress scores for the second/third trimesters since the effect of mid- and late pregnancy on offspring behavior was found to be the largest (23). The items were coded dichotomously based on the presence of experience. The score range is 0-11, with higher scores indicating higher prenatal maternal stress.

**Prenatal Psychosomatic Complaints (T1).** We used the Zerssen List (Beschwerden-Liste) to measure psychosomatic complaints of mothers (24), demonstrating high internal consistency ranging from 0.87 to 0.95. Parallel-test correlations between the two scales were strong (r = 0.85 in a normative sample, N = 1,761; r = 0.88 in a psychiatric comparison group, N = 379) (25). The list included 29 items with four-level rating (1=not at all, 2=hardly, 3=moderate, 4=strong). Symptoms in the last year were measured, covering pregnancy and new-born period. The score

range in the current sample is 24-96, with higher scores indicating higher maternal psychosomatic complaints.

**Maternal Stimulation (T1).** Videotapes of a 10-min standardized nursing and playing situation between mothers and their 3-month-olds were recorded and evaluated by trained raters using a modified version of the categorical system for micro-analysis of the early mother-child interaction (26). Raters were blind to parental and child risk status. They coded the presence or absence of nine measures of mother-infant interaction behavior (i.e., rating of vocal, facial, and motor responses for maternal stimulation, maternal responsiveness, and infant responsiveness) in 120 five-second intervals. Maternal stimulation included all attempts (vocal, facial or motor) to attract the infant´s attention or to establish contact with the infant. It was coded as present when the baby was gazing at the mother or the behaviors were directed to the child. The scores were z-transformed, and recoded such that higher scores indicated lower maternal stimulation.

The observational coding system demonstrated high inter-rater reliability (rs = 0.66–0.97; mean rs = 0.88) and good to excellent internal consistency for maternal sensitivity (α = 0.84) in the original validation study (27).

**Family Adversity (T1-T4).** Family adversity scores were calculated based on the presence of 11 adverse family factors covering characteristics of the parents, the partnership, and the family environment (5). The adverse family factors included low education of a parent, overcrowding, parental psychopathology, parental broken home, marital discord, early parenthood, one-parent family, unwanted pregnancy, poor social integration, severe chronic life difficulties, and poor coping skills of a parent. Early parenthood and unwanted pregnancy were measured only at T1. Inter-rater reliability was excellent for most factors (κ = 0.93–1.0) and moderate for social integration (κ = 0.71) and coping skills (κ = 0.67) (28). To cover adverse family factors occurring in early life (T1-T4), we calculated sum score for each item. Since unwanted pregnancy and early parenthood were measured only once, their score range is 0-1. The score

range for the items measured across the four assessment waves is 0-4, with higher scores indicating chronicity (i.e., multiple occurrence of an adverse factor across the time).

**Income-to-Needs Ratio (T1 and T5).** To calculate income-to-needs ratio, we first determined household income per person by dividing the total household net income to the weighted number of household members, as defined by the Organization for Economic Co-Operation and Development (29). The weighting system assigns a value of 1 to the first adult, 0.7 to each additional adult, and 0.5 to each child. After calculating the household income per person, we divided this value by the national net income. Ratios below one indicate that household income is below the national average. The income-to-need ratio was calculated at T1 and T5, and the scores were log-transformed to normalize data distribution.

**Parental Daily Alcohol Consumption (T1-T6).** Alcohol consumption of parents was recorded between T1 (3 months) and T6 (age 15) using standardized interviews. Parents were asked about their daily alcohol consumption (mg) in the last 4 weeks. We constructed a new continuous variable reflecting the minimum daily alcohol consumption of a parent for the time period up to 15 years.

**Parental Alcohol Misuse/Abuse (T1-T6).** Parents underwent diagnostic interviews between T1 (3 months) and T6 (age 15). Between T1 and T3, alcohol misuse or abuse was determined via the standardized interview. Higher daily alcohol consumption (> 80 mg), speech/motor problems due to alcohol use (at least once a week) and drinking before the noon were among the diagnostic criteria. Between T4-T6, parental alcohol misuse/abuse was noted if a participant had at least one parent diagnosed with alcohol dependency or misuse based on the German version of structured clinical interview for DSM (30).

**Bullying (T5).** Participants were asked about their peer relationships at the age of 11. Bullying was noted if the participant was teased, hit, or nicknamed by another peer.

**Peer Rejection (T5).** Function level of children was measured using the Function Level Scale (31).The peer relationships subscale was used to calculate peer rejection. The subscale had higher inter-rater reliability (r=0.83) (32). The participants having scores equal to three or lower were classified as peer-rejected. Peer-rejected children (score=3) had limited and variable popularity, had a few and frequently changing friends, and were rejected by most of their peers. In extreme cases (score < 3), they had no friend or contact.

**Deviant Peers (T6).** At the age of 15, participants reported whether they have friends who are smoking, consuming alcohol, using drugs, stealing/beating up someone, skipping school, being expelled from school, or having trouble with the police. The score range is 0-7.

**Early alcohol onset (T7).** Participants self-reported the age they consumed a glass of alcohol for the first time. A dummy-coded variable was created to represent early alcohol onset. The variable is coded 1 when the participant started alcohol before the age 15(33).

**Childhood Trauma Questionnaire (T9).** Childhood Trauma Questionnaire (CTQ) is a self-reported measure of traumatic experiences that occurred in childhood(34). We here used the German version of CTQ (35) which includes 28 items covering five subscales: emotional abuse, emotional neglect, physical abuse, physical neglect, and sexual abuse. Responses are quantified on a 5-point Likert scale (1= ‘never true’, 5= ‘very often true’). The German version of the CTQ has demonstrated good reliability and validity, with confirmatory factor analyses replicating the established factor structure, high internal consistency for most scales (Cronbach’s α ≥ 0.89), and low-to-moderate correlations with related measures of psychopathology, comparable to the original English version (35). The total score range is 25-87 in the current sample, with higher scores indicating higher trauma exposure. We here used total CTQ scores (M=31.35, SD=9.06).

**Stressful Life Events (T1-T11).** We measured stressful life events from the first assessment wave (age of 3 months) until the last assessment wave (age of 33 years) using a modified version of the Munich Event List (MEL) (36). The MEL is an interview procedure for assessing acute

and chronic as well as positive and negative stressors. It covers items regarding parental socio-economic disadvantages, negative health outcomes, and living and environmental conditions. Test–retest reliability of the interview was high, with an overall agreement of 95.49% (κ = 0.85), stable concordance across recent and more distant time periods (κ = 0.83–0.84), strong accuracy in dating incidents, and evidence that recall was more reliable for severe than for less salient events, despite a modest long-term fall-off effect (37).

Parents rated the MEL up to the sixth assessment wave (age 15). From the 15-year assessment onwards, participants rated stressful life events themselves. Here, we used total scores from T1 (3 months) to time T6 (15 years), which reflected total stress exposure at each assessment wave. Higher scores indicated exposure to more stressful life events.

# Other Measures

**Early Behavioral Symptoms (T1-T3).** At the age of 3 months (T1), we assessed only a small area of infant behavior including feeding and sleeping (38). In addition, behavioral problems of 3-month-olds were assessed in terms of 13 adverse temperamental characteristics such as irritability, distractibility, or dysphoric mood, which were derived from the nine temperament scales (39). At the ages of 2 (T2) and 4.5 (T3) years, a broad range of symptoms was evaluated comprising temper tantrums, hyperactivity, attentional deficits, oppositional behavior, separation anxiety, social inhibition, feeding and sleeping problems, sibling rivalry, tics, and stereotypes (38). The behavioral problems lists included 15, 35, and 29 items at T1, T2, and T3, respectively. The item ratings ranged between 0-2 (0= not affected, 1= light symptoms, 2= clearly affected). Inter-rater reliability of two raters on single behaviour problems averaged kappa = 0.68 at 3 months and kappa = 0.83 at 2 and 4.5 years (38).

**Intelligent Quotient (T5).** To calculate intelligent quotient (IQ) scores, we used German version of the Culture Fair Test non-verbal intelligence (40) (non-verbal IQ) and vocabulary test

(41) (verbal IQ), which are well-established intelligence measures with documented high reliability and good validity. Standardized IQ scores (mean=100) were used in the current study.

**Temperament (T4 and T6).** Between T1 (3 months) and T5 (age 11), we assessed temperamental characteristics in two ways: within the frame of a standardized parent interview and during structured direct observations in 4 standardized settings on 2 different days in both familiar (home) and unfamiliar (laboratory) surroundings (42). All ratings were made by trained judges on five-point rating scales of nine temperamental dimensions adapted from the New York Longitudinal Study (39). Satisfactory inter-rater reliability was observed in a preliminary study, assessed at 3 months (κ = 0.68) and 2 years (κ = 0.82) in subsamples of participants (N = 30 at each time point) (42).

The dimensions consisted of (1) activity, as the frequency and intensity of motor behavior; (2) rhythmicity, referring to the regularity in biological functions; (3) approach/withdrawal, describing the initial reaction to new stimuli (e.g., strangers or new food);

(4) adaptability, as the length of time that is needed to be habituated to the new stimuli; (5) threshold of responsiveness, as the sensitivity in the child's reaction to a range of external stimuli or environmental changes (e.g., temperature of the food, pain); (6) intensity of reaction, referring to the expression of positive and negative emotions; (7) the prevailing mood on a continuum between positive and negative mood; (8) distractibility/soothability, as the ease in which the child is soothed after unpleasant occurrences such as disappointments, failures, or a trifling physical pain; and (9) attention span/persistence, referring to the child's ability to pursue a particular activity and its continuation in the face of obstacles (42,43). At each assessment, the principal component analysis of the nine temperamental dimensions produced a 2-factor solution (42,43). The first factor resembled the easy-difficult trait. Primary loadings were distractibility/soothability, mood, approach/withdrawal, and adaptability. The second temperament factor resembled the self-control, which had positive loadings of

attention/persistence and distractibility/soothability, and negative loadings of activity and intensity. To reduce the number of features, we only used the measures at T4, which characterized the childhood temperament at baseline.

At age 15 (T6), we used temperament measures from German version of the Junior Temperament and Character Inventory (JTCI) (44). The inventory included four subscales for temperament: Novelty seeking, harm avoidance, reward dependence, and persistence. The German version of the JTCI shows overall satisfactory psychometric properties, including acceptable internal consistency for most scales and good construct validity, with factor analyses supporting the original temperament and character structure (44). Total scores from the four subscales were used in the current study.

**Function Level (T4 and T5).** Function level was measured at T4 (age 8) and T5 (age 11) using Function Level Scale (31). At T4, Function Level Scale included four dimensions: Family, peer relationships, interests, and autonomy. At T5, the scale included an additional dimension: School performance. Trained interviewers rated functionality for each dimension. Dimensional scores ranged between one and seven, with higher scores indicated higher functionality. The scale had high inter-rater reliability (r= 0.83-0.92), and the subscales showed moderate correlations with mental health status (32).

**Self-Perception (T4 and T6).** Self-perception was measured at age 8 (T4) and 15 (T6) using the German version of the Self-Perception Scale for Children, demonstrated good psychometric properties (45). The measure included 23 items with three subscales at T4: Scholastic competence, social competence, and athletic competence. Each item represented a skill on a 4-point Likert scale. T6 measure included 25 items with five subscales: Scholastic competence, social competence, physical appearance, behavioral conduct, and global self-worth. Each item included two opposing statements (e.g., statement 1: *“Some youth have difficulty in making*

*friends.”*; statement 2: *“It is easy to make friends for other youths.”*). Participants chose the statement fitting them and rated it as “sort of true for me” or “really true for me”.

**Table S7.** Feature list including measures collected up to age 15.

| **Feature** | **Measurement Time** | **N** | **Range** | **Mean (SD) or n (%)** | **Brief Description** |
| --- | --- | --- | --- | --- | --- |
| Sex | T1 | 317 | 0-1 | 171 (53.9%)  female | Biological sex at birth (0=male, 1=female) |
| PGS ADHD | T6 | 298 | -0.0004 –  0.0004 | 0.0000  (0.0001) | Polygenic risk score for attention deficit and hyperactivity disorder |
| PGS AG | T6 | 298 | -0.0437-  0.0347 | 0.0001  (0.0135) | Polygenic risk score for aggression |
| PGS Alcohol | T6 | 298 | -0.0145 –  0.0146 | 0 (0.0049) | Polygenic risk score for alcohol dependence |
| PGS Anxiety | T6 | 298 | -0.0003 –  0.0004 | 0 (0.0001) | Polygenic risk score for anxiety |
| PGS MDD | T6 | 298 | -0.0001 –  0.0001 | 0 (0.00003) | Polygenic risk score for major depressive disorder |
| PGS SCZ | T6 | 298 | -0.0003 –  0.0004 | 0 (0.0001) | Polygenic risk score for schizophrenia |
| PGS CP | T6 | 298 | -0.0001 –  0.0001 | 0 (0.00002) | Polygenic score for cognitive performance |
| PGS EA | T6 | 298 | -0.00004-  0.00004 | 0 (0.00001) | Polygenic score for educational attainment |
| PGS IQ | T6 | 298 | -0.0001 –  0.0001 | 0 (0.00003) | Polygenic score for intelligent quotient |
| PGS NCEA | T6 | 298 | -0.0002 –  0.0002 | 0 (0.00006) | Polygenic score for non-cognitive educational attainment |
| Obstetric risk | T1 | 317 | 0-4 | 1 (1) | Birth complications, preterm birth, low birth weight |
| Moderate maternal smoking | T1 | 317 | 0-1 | 31 (9.8%) | Prenatal maternal smoking: Daily consumption up to 5 cigarettes |
| Heavy maternal smoking | T1 | 317 | 0-1 | 48 (15.1%) | Prenatal maternal smoking: Daily consumption more than 5 cigarettes |
| Maternal stress | T1 | 317 | 0-10 | 3 (2.1) | Sum of 11 items covering negative experiences during second or third trimester of pregnancy |
| Maternal psychosomatic complaints | T1 | 317 | 28-84 | 46.3 (10) | Mother’s psychological and somatic complaints in the last year |
| Income-to-needs ratio | T1 | 317 | -0.8 – 0.8 | 0.2 (0.2) | Economical well-being of family compared to national average (Log-transformed scores) |
| Lower maternal | T1 | 304 | -8.2 – 7.2 | 0.02 (2.5) | Mothers’ attempts (vocal, facial or motor) to draw |

| stimulation |  |  |  |  | infants’ attention (video-recorded) [z-transformed reversely coded scores] |
| --- | --- | --- | --- | --- | --- |
| Low parental education | T1-T4 | 315 | 0-4 | 0.7 (1.4) | Parent without educational qualifications or skilled job training |
| Overcrowding | T1-T4 | 315 | 0-4 | 0.2 (0.6) | Less than one room per person in the home or home size < 50 m2 |
| Parental psychopathology | T1-T4 | 315 | 0-4 | 1.1 (1.3) | Moderate or severe disorder according to DSM-III-R criteria |
| Parental broken home | T1-T4 | 315 | 0-4 | 0.7 (1.4) | Institutional care of a parent/more than 2 changes of parental figures until the age of 18 or history of parental delinquency |
| Marital discord | T1-T4 | 315 | 0-4 | 0.7 (1.1) | Low quality of partnership in 2 out of 3 areas (harmony, communication, emotional warmth) |
| Unwanted pregnancy | T1 | 317 | 0-1 | 46 (14.5%) | An abortion is seriously considered |
| One parent family | T1-T4 | 315 | 0-4 | 0.5 (1.1) | One parent family / single parent |
| Early parenthood | T1 | 317 | 0-1 | 82 (25.9%) | Age of a parent ≤18 years at child’s birth or relationship between parents lasting <6 months at time of conception |
| Poor social integration | T1-T4 | 315 | 0-4 | 0.3 (0.7) | Lack of friends/of support in everyday life |
| Severe chronic events | T1-T4 | 315 | 0-4 | 1.2 (1.1) | Affecting a parent lasting >1 year |
| Poor coping skills - Parents | T1-T4 | 315 | 0-4 | 1.7 (1.5) | Inadequate coping with stressful events of the past year |
| Stressful life events | T1 | 317 | 0-15 | 3.8 (2.4) | Total stress exposure scores (prenatal-3 months) |
| Stressful life events | T2 | 316 | 0-21 | 5.4 (3) | Total stress exposure scores (3 months-2 years) |
| Stressful life events | T3 | 317 | 1-22 | 7 (3.5) | Total stress exposure scores (2 years-4.5 years) |
| Stressful life events | T4 | 316 | 0-23 | 6.9 (3.6) | Total stress exposure scores (4.5 years-8 years) |
| Behavioral symptoms | T1 | 317 | 0-11 | 2.1 (2.2) | Total behavioral symptoms at T1, covering sleeping, eating problems, irritability etc. |
| Behavioral symptoms | T2 | 316 | 0-19 | 4.4 (3.9) | Total behavioral symptoms at T2 |
| Behavioral symptoms | T3 | 317 | 0-18 | 4.1 (3.5) | Total behavioral symptoms at T3 |
| Temperament- Easy-difficult trait | T4 | 315 | -3.1- 2.1 | 0.02 (1) | Temperament factor reflecting distractibility/ soothability, mood, approach/withdrawal, and adaptability domains |
| Temperament- Self-control | T4 | 315 | -3.9 -1.9 | 0.1 (0.9) | Temperament factor reflecting persistence and distractibility/soothability domains |

| Function level-Family | T4 | 316 | 2-7 | 4.6 (0.9) | Evaluation of child functioning in the family |
| --- | --- | --- | --- | --- | --- |
| Function level- Peers | T4 | 316 | 2-7 | 4.6 (0.9) | Evaluation of peer relationships (e.g., popularity, position in the hierarchy) |
| Function level-Interests | T4 | 316 | 2-7 | 4.7 (0.9) | Evaluation of iversity of interets and free time activities |
| Function level-Autonomy | T4 | 316 | 2-7 | 4.7 (1) | Evaluation of mastery in developmentally assigned tasks |
| Self-perception-Scholastic competence | T4 | 315 | 8-24 | 19.3 (2.9) | Children’s perception of their academic and school performance |
| Self-perception-Social competence | T4 | 315 | 7-24 | 18.3 (3.3) | Children’s perception about their social skills and peer relationships |
| Self-perception-Athletic competence | T4 | 315 | 12-24 | 20.3 (2.5) | Children’s perception of physical ability and performance in sports or outdoor games |
| Testosterone | T6 | 239 | 8-607 | 117.1  (125.5) | Plasma testosterone level (baseline) |
| DHT | T6 | 239 | 1-90 | 17.2 (13.1) | Plasma dihydrotestosterone level (baseline) |
| Cortisol | T6 | 229 | 0-11.9 | 3 (2.3) | Plasme cortisol level (baseline) |
| Peer rejection | T5 | 313 | 0-1 | 32 (10.1%) | Poor functioning in peer relationships (function level <= 3) |
| Bullying | T5 | 305 | 0-1 | 72 (22.7) | Being teased, hit or nicknamed by a peer |
| Income-to-needs ratio | T5 | 317 | 0.38- 0.73 | 0.2 (0.2) | Economical well-being of family compared to national average (Log-transformed scores) |
| IQ | T5 | 317 | 67.5-  148.8 | 102.9 (14.8) | Mental development index at age 11 |
| Stressful life events | T5 | 316 | 0-18 | 5.4 (3.4) | Total stress exposure scores (8 years-11 years) |
| Stressful life events | T6 | 307 | 0-22 | 6.2 (3.6) | Total stress exposure scores (11 years-15 years) |
| Deviant peers | T6 | 312 | 0-7 | 2.1 (1.9) | Having peers who engage in behaviors that deviate from social norms (e.g., stealing, using drugs, smoking) |
| Early alcohol onset | T6-T7 | 317 | 0-1 | 247 (77.9%) | Consuming a glass of alcohol before the age of 15 |

| Parental alcohol misuse/abuse | T1-T6 | 314 | 0-1 | 56 (17.7%) | Having a parent diagnosed with alcohol dependence or misuse |
| --- | --- | --- | --- | --- | --- |
| Parental alcohol consumption | T1-T6 | 317 | 0-10 | 2 (2.1) | Minimum daily alcohol consumption of a parent |
| Childhood Trauma Questionnaire | T9 | 258 | 25-87 | 31.3 (9.1) | Self-reported retrospective measure of abuse and neglect in the first 18 years (total scores) |
| Temperament-Novelty seeking | T6 | 315 | 0-36 | 20.1 (6.6) | Tendency of excitement toward a new stimuli (exploratory behavior, impulsivity, extravagance, lack of discipline) |
| Temperament- Harm avoidance | T6 | 315 | 0-35 | 15.5 (6.7) | Propensity to avoid harm (anticipatory worry, fear of uncertainity, shyness, fatigability) |
| Temperament-Reward dependence | T6 | 315 | 0-29 | 17.2 (4.4) | Sensitivity to social rewards and attachment to others |
| Temperament-Persistence | T6 | 315 | 0-24 | 14.1 (4.2) | Perseverance despite frustration and fatigue |
| Function level-Family | T5 | 316 | 2-6 | 4.7 (0.8) | Evaluation of child functioning in the family |
| Function level- Peers | T5 | 316 | 2-6 | 4.6 (0.9) | Evaluation of peer relationships (e.g., popularity, position in the hierarchy) |
| Function level-Interests | T5 | 316 | 2-7 | 4.7 (0.9) | Evaluation of diversity of interets and free time activities |
| Function level-School performance | T5 | 316 | 2-7 | 4.8 (0.9) | Evaluation of academic achievement in major subjects |
| Function level-Autonomy | T5 | 311 | 2-7 | 4.8 (1) | Evaluation of mastery in developmentally assigned tasks |
| Self-perception-Scholastic competence | T6 | 316 | 0-20 | 14.2 (3.1) | Children’s perception of their academic and school performance |
| Self-perception-Social competence | T6 | 316 | 0-20 | 15.9 (3.8) | Children’s perception about their social skills and peer relationships |
| Self-perception-Physical competence | T6 | 316 | 0-20 | 13.8 (3.9) | Children’s perception about their physical looks and attractiveness |
| Self-perception-Behavioral conduct | T6 | 316 | 0-20 | 14.8 (3.4) | Self-perception Scale for Children- Total scores in physical competence subscale |
| Self-perception-Global self-worth | T6 | 316 | 0-20 | 15.5 (3.5) | Self-perception Scale for Children- Total scores in physical competence subscale |

T1: 3 months, T2: 2 years, T3: 4.5 years, T4:8 years, T5:11 years, T6: 15 years, T9:25 years.

# Appendix S4. Machine Learning Pipeline

The analysis pipeline included missing value imputation, scaling, model estimation with hyperparameter tuning, and model evaluation. IterativeImputer and the most common category were used to impute missing values for numerical and categorical (i.e., dummy-coded) data, respectively. Continuous data were then scaled using MinMaxScaler. We used RandomizedSearchCV for hyperparameter tuning. Class imbalance was handled using the parameter “class_weight”. Table S8 shows hyperparameters settings for each model.

**Table S8.** Hyperparameter Settings.

| **Model** | **Hyperparameter** | **Parameter Space** |
| --- | --- | --- |
| Logistic Regression | solver | ['lbfgs','liblinear', 'newton-cg'] |
|  | class_weight | ['balanced', None] |
|  | C | np.logspace(-4,4,50) |
| Support Vector Machines | kernel | ['linear', 'rbf', 'poly'] |
|  | C | np.logspace (-4,4,50) |
|  | gamma | ['scale', 'auto'] + list(np.logspace(-3, 3, 50)) |
|  | class_weight | ['balanced', None] |
|  | decision_function | ['ovo','ovr'] |
| Random Forest | N_estimators | [int(x) for x in np.linspace(start = 50, stop = 1000, num = 100)] |
|  | max_features | ['log2', 'sqrt'] |
|  | max_depth | sp_randint (2,10) |
|  | min_samples_split | sp_randint (2,20) |
|  | min_sample_leaf | sp_randint (1,20) |
|  | class_weight | ['balanced', None] |
| XGBoost | learning_rate | sp_randfloat(0.01,1) |
|  | max_depth | sp_randint(1,10) |
|  | min_child_weight | sp_randint(1,10) |
|  | subsample | np.arange(0.5, 1, 0.1) |
|  | objective | ['multi:softmax', 'multi:softprob'] |

Notes: np=import numpy as np; sp_randfloat= from scipy.stats import uniform as sp_randfloat; sp_randint =from scipy.stats import randint as sp_randInt

# Appendix S5. Measures for Supplemental Analyses

We conducted series of supplemental analysis to understand symptom changes across development. Chi-square test was used for categorical outcomes. We used one-way ANOVA test for numerical outcomes. Kruskal-Wallis Test was used instead when the assumptions were violated.

# Adult Functioning

We used several measures of adult functioning at T11 (33 years).

# 1. Education

***School Degree (T6-T11).*** Participants were asked about their education status in multiple assessment waves. Based on their responses, we constructed five school degree categories compatible with German academic system: no degree, secondary school (Hauptschule), intermediate school (Mittlere Reife), vocational school (Fachabitur), and high school (Abitur).

***Higher Education (T7-T11).*** This measure is consisted of four categories: no degree, student, vocational training (Ausbildung), and university degree.

2. **Income (T11).** We calculated monthly net income per person using the criteria from the Organization of Economic Co-operation and Development modified scale [household income/(adult number *0.5+ child number*0.3+1)]

# 3. Physical Health (T11)

***Body Mass Index.*** Body mass index was calculated based on participant’s weight and height at the last assessment wave.

***Chronic illness.*** Participants were asked if they had a chronic illness (yes/no).

***Hospital Admission.*** Participants were asked if they had hospital admission in the last year (e.g., due to accidents, surgery etc.).

# 4. Life Time Mental Health Diagnosis (T3-T11)

Participants underwent diagnostic interviews from T3 (age 4.5) on. We created a dichotomous variable and coded with one if a participant had mental health diagnosis between T3 and T11 based on DSM criteria (DSM III-R, DSM-IV or DSM V).

# 5. Substance Use (T11)

At T11, participants self-reported their smoking and cannabis use status (yes or no). Alcohol consumption was measured using Alcohol Use Identification Test (46). The German version of the AUDIT has demonstrated excellent test–retest reliability for the total score (ICC = 0.95), good agreement at commonly used cut-off values (κ = 0.81–0.86), and strong validity, with high sensitivity and specificity for identifying at-risk drinking and alcohol use disorders in primary care settings (47). We used total scores as a measure of alcohol use.

# 6. Social Functioning (T11)

At T11, participants were asked about their partnership status (yes/no), if they live alone (yes/no), number of friends (none, 1, 2-3, 4 and more), monthly friend contact (less than a month, 1-2 times, 3-4 times, 5 and more), and friendship quality.

# Clinical and Psychosocial Correlates of Symptom Change

1. **Treatment**

Between T2 (age 2) and T7 (age 19), we measured if participants received any kind of treatment due to their behavioral problems via the standardized interviews. Between T8 (age 22) and T11 (age 33), participants self-reported if they used any medication or receive a psychotherapy due to their psychological problems. We compared the trajectory groups to see if they differ in terms of receiving treatment using Chi-Square test. With this analysis, we aimed to discover if symptom decreases can be explained by early treatment access or increasing symptoms might

be related to not receiving any treatment. We did not conduct any further analyses (e.g., treatment type, duration, success) since only a small portion of participants received treatment.

# 2. Stressful Life Events

We measured stressful life events from the first assessment wave (age of 3 months) until the last assessment wave (age of 33 years) using a modified version of the Munich Event List (MEL)

(36). The MEL is an interview procedure for assessing acute and chronic (> 3months) as well as positive and negative stressors. Items cover several domains, including parental socio-economic disadvantages, negative health outcomes, and living and environmental conditions. In addition, the items included both normative (i.e., expected life changes during the life course, such as school and job transitions, marriage, childbirth, moving) and non-normative (i.e., unexpected life changes, such as loss of loved ones, separation, hospitalization, incarceration) stressors. Test–retest reliability of the interview was high, with an overall agreement of 95.49% (κ = 0.85), stable concordance across recent and more distant time periods (κ = 0.83–0.84), strong accuracy in dating incidents, and evidence that recall was more reliable for severe than for less salient events, despite a modest long-term fall-off effect (7).

Parents rated the MEL up to the sixth assessment wave (age of 15 years). From the 15-year assessment onwards, participants rated stressful life events themselves. Scores reflected total stress exposure. Higher scores indicated more exposure to stressful life events.

# 3. Coping Skills

At T4 (age 8) and T5 (age 11), trained interviewers rated coping skills of participants for daily and stressful situations. The score ranged between one and seven, with higher scores indicated higher coping skills. At T8 (age 22), we used short form of the Stress Coping Style Questionnaire (48). The questionnaire included 13 positive and negative strategies: Play down, guilt denial, substitutional satisfaction, situation control, reaction control, positive self-instruction, need for social support, active avoidance, flight tendency, rumination, resignation, and self-accusation. The measure has demonstrated good internal consistency and reliability (Cronbach’s α = 0.77–0.93; split-half reliability r = 0.74–0.95; test–retest reliability r = 0.61–0.74), along with evidence of solid construct and external validity (49,50). We here focused on average scores from three positive dimensions, devaluation (playing down, guilt denial), distraction (distraction, substitutional satisfaction), and control (situation control, reaction control, positive self-instruction), and negative strategies (flight tendency, rumination, resignation, and self-accusation) (51).

# 4. Resources

At T4 (age 8) and T5 (age 11), social support and integration of participants were rated by trained interviewers. The score ranged between one and seven, with higher scores indicated higher social support and integration. At T10 (age 25), we measured personal and social resources of individuals using the Resources Checklist (52). In the current study, we used scores from six domains: partner, friends and family, home, work, leisure, and religion. We were unable to identify empirical studies that have formally assessed the reliability and validity of this measure.

# Appendix S6. Extended Results for Trajectory Modeling

# Group Mean

Mean symptom trajectories for internalizing and externalizing symptoms were visualized in Figure S1. Across development, internalizing symptoms showed an increasing trajectory, peaked around young adulthood (age 19) and then decreased slightly, whereas externalizing symptoms peaked around adolescence (age 15) and decreased significantly afterwards.

**Figure S1.** Mean Trajectories for Internalizing and Externalizing Symptoms across Development.

We identified similar trajectories for the subscales constituting internalizing and externalizing symptom scores (Figure S2). For internalizing problems subscales (i.e., anxious-depressed, somatic complaints, withdrawn), symptom scores showed increasing trajectory and peaked around the age of 19 (i.e., young adulthood). For externalizing problems subscales (aggressive behavior, intrusive, rule-breaking behavior), the symptom scores peaked around the

age of 15 (i.e., adolescence) and decreased afterwards. Since the intrusive subscale was available from 19 years on and included items that were previously asked under the aggression subscale, aggressive behavior and intrusive behavior were visualized together by simply adding the intrusive subscale scores to the aggressive behavior scores from 19 years on.

**Figure S2.** Mean Symptom Trajectories for the Subscales.

# Trajectory Modeling with Latent Growth Mixture Modeling Internalizing Symptoms

We identified a three-class solution for internalizing symptom trajectories: Low symptoms class (79.5%), increasing symptoms class (13.6%), and decreasing symptoms class (6.9%) (Table S9). The three-class solution had high entropy (> 0.80). Log-likelihood tests indicated that a three-class solution had a significantly better fit than the two-class solution (p <0.05). None of the classes included less than 1% of participants.

**Table S9.** Model fit indices of latent growth mixture models for internalizing symptoms.

| Class | AIC | BIC | Entropy | Class Membership, N(%) | LRT | Adjusted  LRT |
| --- | --- | --- | --- | --- | --- | --- |
| 1-class | -4999 | -4950 | - | - | - | - |
| 2-class | -5070 | -5010 | 0.88 | 49(15.5%), 268(84.5%) | 0.011 | 0.013 |
| **3-class** | **-5119** | **-5048** | **0.88** | **43(13.6%), 22(6.9%), 252(79.5%)** | **0.035** | **0.041** |
| 4-class | -5121 | -5046 | 0.89 | 15(4.7%), 10(3.2%), 240(75.7%),  52(16.4%) | 0.23 | 0.25 |
| 5-class | -5131 | -5044 | 0.76 | 45(14.2%), 60(18.9%), 19(6%),  183(57.7%), 10(3.2%) | 0.45 | 0.46 |

Abbreviations: AIC, Akaike Information Criterion; BIC, Bayesian Information Criterion; LRT, likelihood ratio test.

# Externalizing Symptoms

Similar to the internalizing symptoms trajectories, we found a three-class solution for externalizing symptoms trajectories: Low symptoms class (80.5%), increasing symptoms class (8.5%), and decreasing symptoms class (11%) (Table S10). The three-class solution had high entropy (> 0.80). Log-likelihood tests indicated that a three-class solution had a significantly better fit than the two-class solution (p <0.05). None of the classes included less than 1% of participants.

**Table S10.** Model fit indices of latent growth mixture models for externalizing symptoms.

| Class | AIC | BIC | Entropy | Class Membership, N(%) | LRT | Adjusted  LRT |
| --- | --- | --- | --- | --- | --- | --- |
| 1-class | -5461 | -5412 | - | - | - | - |
| 2-class | -5497 | -5445 | 0.92 | 289(91.2%), 28(8.8%) | 0.0001 | 0.0001 |
| **3-class** | **-5552** | **-5488** | **0.85** | **255(80.5%), 27(8.5%), 35(11%)** | **0.014** | **0.017** |
| 4-class | -5568 | -5492 | 0.85 | 244(77%), 30(9.5%), 27(8.5%),  16(5%) | 0.45 | 0.46 |
| 5-class | -5586 | -5500 | 0.87 | 26(8.2%), 29(9.1%), 236(74.4%),  1(0.03%), 25 (7.9%) | 0.11 | 0.12 |

Abbreviations: AIC, Akaike Information Criterion; BIC, Bayesian Information Criterion; LRT, likelihood ratio test.

# Overlap between Symptom Trajectories

We performed LGMM for internalizing and externalizing symptoms separately. Despite using the sample, using different measures for trajectory modeling will result in different combinations of classes across two symptom domains. Figure S3 illustrates class overlap between internalizing and externalizing problems trajectories. As seen, the majority of participants (n=219) had lower symptom scores in both problem domains, although we observed all possible class combinations in this sample.

**Figure S3.** Overlap between symptom trajectories.

# Group Comparisons using Symptom Scores

We compared the trajectory groups using the scaled symptom scores (i.e., proportion of maximum scaled) for externalaizing and internalizing problems. Externalizing and internalizing symptoms within each group were not normally distributed. Therefore, we used Kruskal-Wallis test, which is a non-paramteric alternative of one-way ANOVA test. If a significant main effect of group was observed, we conducted post-hoc group comparisons. Post-hoc tests were corrected for multiple testing using Bonferroni correction.

**Table S11.** Comparisons of externalizing symptoms trajectories in terms of externalizing symptoms.

| **Measure** | **Increasing Symptoms**  **Median (IQR)** | **Decreasing Symptoms**  **Median (IQR)** | **Low Symptoms Median (IQR)** | **df** | **Test statistics** | **p** |
| --- | --- | --- | --- | --- | --- | --- |
| T4 | 0.09 (0.12) | 0.22 (12) | 0.09 (0.11) | 2 | 33.56 | **0.000***** |
| T5 | 0.08 (0.12) | 0.19 (0.13) | 0.06 (0.10) | 2 | 18.53 | **0.000***** |
| T6 | 0.20 (0.17) | 0.13 (0.14) | 0.15 (0.12) | 2 | 15.33 | **0.000***** |
| T7 | 0.20 (0.18) | 0.11 (0.17) | 0.09 (0.14) | 2 | 31.14 | **0.000***** |
| T8 | 0.20 (0.20) | 0.06 (0.16) | 0.07 (0.08) | 2 | 32.7 | **0.000***** |
| T9 | 0.16 (0.09) | 0.04 (0.12) | 0.07 (0.09) | 2 | 29.4 | **0.000***** |
| T10 | 0.14 (0.20) | 0.04 (0.10) | 0.05 (0.09) | 2 | 26.6 | **0.000***** |
| T11 | 0.20 (0.17) | 0.06 (0.09) | 0.06 (0.08) | 2 | 30.83 | **0.000***** |

**Table S12.** Post-hoc tests for group comparisons: externalizing symptoms trajectories.

|  | **Low vs. Increasing**  **Symptoms** | | **Low vs. Decreasing**  **Symptoms** | | **Increasing vs. Decreasing**  **Symptoms** | |
| --- | --- | --- | --- | --- | --- | --- |
|  | Test  Statistics | p | Test Statistics | p | Test  Statistics | p |
| T4^a^ | -29.7 | **0.12** | -113.3 | **0.000***** | -83.6 | **0.001**** |
| T4^a^ | -14.9 | 0.94 | -88.6 | **0.000***** | -73.7 | **0.007**** |
| T6^a^ | -58.1 | **0.000**** | -16.8 | 1 | 41.3 | 0.25 |
| T7^a^ | -81.2 | **0.000***** | -40.4 | 0.14 | 40.8 | 0.26 |
| T8^a^ | -84.5 | **0.000***** | - 6.1 | 1 | 90.6 | **0.000***** |
| T9^a^ | -80.4 | **0.000***** | -7.2 | 1 | 73.1 | **0.006**** |
| T10^a^ | -76.3 | **0.000**** | -6 | 1 | 82.3 | **0.001**** |
| T11^a^ | -75.4 | **0.000*** | -5 | 1 | 80.4 | **0.001**** |

^a^ Kruskal-Wallis Test

**Table S13.** Group comparisons of internalizing symptoms trajectories in terms of internalizing symptoms.

| **Measure** | **Increasing Symptoms**  **Median (IQR)** | **Decreasing Symptoms**  **Median (IQR)** | **Low Symptoms Median (IQR)** | **df** | **Test statistics** | **p** |
| --- | --- | --- | --- | --- | --- | --- |
| T4 | 0.03 (0.07) | 0.15 (0.10) | 0.06 (0.09) | 2 | 28.73 | **0.000***** |
| T5 | 0.04 (0.06) | 0.18 (0.16) | 0.06 (0.08) | 2 | 35.7 | **0.000***** |
| T6 | 0.16 (0.11) | 0.09 (0.09) | 0.10 (0.10) | 2 | 7.86 | **0.02*** |
| T7 | 0.25 (0.10) | 0.11 (0.12) | 0.12 (0.16) | 2 | 15.9 | **0.000***** |
| T8 | 0.25 (0.28) | 0.10 (0.10) | 0.11 (0.12) | 2 | 17.8 | **0.000***** |
| T9 | 0.21 (0.18) | 0.09 (0.09) | 0.08 (0.11) | 2 | 23.05 | **0.000***** |
| T10 | 0.26 (0.23) | 0.07 (0.13) | 0.09 (0.13) | 2 | 23.4 | **0.000***** |
| T11 | 0.23 (0.22) | 0.05 (0.11) | 0.09 (0.15) | 2 | 17.76 | **0.000***** |

**Table S14.** Post-hoc tests for group comparisons: internalizing symptoms.

|  | **Low vs. Increasing**  **Symptoms** | | **Low vs. Decreasing**  **Symptoms** | | **Increasing vs. Decreasing**  **Symptoms** | |
| --- | --- | --- | --- | --- | --- | --- |
|  | Test  Statistics | p | Test Statistics | p | Test  Statistics | p |
| T4 ^a^ | -3.4 | 1 | -84.6 | **0.000***** | -88 | **0.000***** |
| T5 ^a^ | -4.9 | 1 | -98.6 | **0.000***** | -94.1 | **0.000***** |
| T6 ^a^ | -44.4 | **0.05** | -27.9 | 0.26 | 16.6 | 1 |
| T7 ^a^ | -69.5 | **0.001**** | -28.5 | 0.26 | 41 | 0.24 |
| T8 ^a^ | -77.1 | **0.000***** | -15.3 | 1 | 61.8 | **0.024*** |
| T9 ^a^ | -87.1 | **0.000***** | -16.3 | 0.99 | 70.8 | **0.007**** |
| T10 ^a^ | -90.3 | **0.000***** | -3.9 | 1 | 86.4 | **0.001**** |
| T11 ^a^ | -59.9 | **0.001**** | -30.55 | 0.17 | 90.45 | **0.000***** |

^a^ Kruskal-Wallis Test

# Appendix S7. Extended Results for Machine Learning Analysis

# Model Performance

Using 73 features collected up to adolescence (15 years), we predicted symptom trajectories for internalizing and externalizing symptoms using four machine learning models: Logistic regression, support-vector machines, random forest and XGBoost. Although random forest and logistic regresstion were the winning models for prediction of externalizing and internalizing symptom trajectories, respectively, other predictive models also showed similar performance (Table S15).

**Table S15.** Model performance metrics for the prediction of symptom trajectories.

| **Externalizing Symptoms Trajectories** | | | | | |
| --- | --- | --- | --- | --- | --- |
| **Model** | **F1-weighted** | **F1-macro** | **Class-specific ROC-AUC** | **Micro ROC-AUC** | **Macro ROC-AUC** |
| Logistic regression | 0.67 | 0.40 | Low: 0.66  Increasing: 0.64  Decreasing: 0.64 | 0.81 | 0.64 |
| Support-vector machines | 0.69 | 0.43 | Low: 0.66  Increasing: 0.66  Decreasing: 0.67 | 0.90 | 0.66 |
| **Random forest** | **0.71** | **0.43** | **Low: 0.74**  **Increasing: 0.77**  **Decreasing: 0.76** | **0.88** | **0.77** |
| XGBoost | 0.72 | 0.38 | Low: 0.64  Increasing: 0.69  Decreasing: 0.69 | 0.89 | 0.67 |
| **Internalizing Symptoms Trajectories** | | | | | |
| **Model** | **F1-weighted** | **F1-macro** | **Class-specific ROC-AUC** | **Micro ROC-AUC** | **Macro ROC-AUC** |
| **Logistic regression** | **0.70** | **0.49** | **Low: 0.73**  **Increasing: 0.71**  **Decreasing: 0.80** | **0.83** | **0.75** |
| Support-vector machines | 0.71 | 0.47 | Low: 0.71  Increasing: 0.70  Decreasing: 0.76 | 0.91 | 0.72 |
| Random forest | 0.70 | 0.44 | Low: 0.72  Increasing: 0.69  Decreasing: 0.82 | 0.86 | 0.75 |
| XGBoost | 0.70 | 0.36 | Low: 0.60  Increasing: 0.55  Decreasing: 0.72 | 0.86 | 0.62 |

# Trajectory Specific Features

We used SHAP (SHapley Additive exPlanations) to quantify how much a given feature increases or decreases the predicted probability of a specific outcome relative to the model’s average prediction. Positive SHAP values indicate that a feature contributes to a higher predicted probability of a given class, whereas negative values indicate a contribution toward a lower predicted probability. The SHAP beeswarm plots (Figures S4-S9) display all 73 features ranked by mean absolute SHAP value in the Y-axis, with higher positions indicating greater overall contribution to the model. The X-axis shows the distribution of SHAP values, where values to the right of zero indicate a positive contribution toward predicting the class and values to the left indicate a negative contribution. Color represents feature values, with blue indicating lower values and pink indicating higher values. For example, higher self-control scores are associated with a greater likelihood of being classified in a low-symptoms tracjectory for externalizing symptoms (Figure S4).

**Figure S4.** Full list of 73 features contributing to model prediction of low symptoms trajectory class for externalizing symptoms.

**Figure S5.** Full list of 73 features contributing to model prediction of increasing symptoms trajectory class for externalizing symptoms.

**Figure S6.** Full list of 73 features contributing to model prediction of decreasing symptoms trajectory class for externalizing symptoms.

**Figure S7.** Full list of 73 features contributing to model prediction of low symptoms trajectory class for internalizing symptoms.

**Figure S8.** Full list of 73 features contributing to model prediction of increasing symptoms trajectory class for internalizing symptoms.

**Figure S9.** Full list of 73 features contributing to model prediction of decreasing symptoms trajectory class for internalizing symptoms.

# Appendix S8. Results for Supplementary Analyses

# Adulthood Outcomes

We found that psychopathology risk trajectories were associated with several adverse adulthood outcomes including lower education, lower income, more hospital admission, a higher life-time prevalence of mental health diagnosis, and poor social functioning (Table S16-S19). Risk trajectories for externalizing disorders additionally had higher substance use (Table S16-S17).

**Table S16.** Comparisons of externalizing symptom trajectories in terms of adult functioning.

|  | **Low Symptoms** | **Increasing Symptoms** | **Decreasing Symptoms** | **Test Statistics** | **df** | **p** |
| --- | --- | --- | --- | --- | --- | --- |
| **Demographics** |  |  |  |  |  |  |
| Sex, N, F/M | 122/89 | 10/11 | 12/12 | 1.23 | 2 | 0.33 |
| School degree, N, no degree/secondary school/ intermediate school/ vocational school/ high school | 1/24/56/18/112 | 0/2/15/0/4 | 0/7/8/1/8 | 26.30 | 8 | **0.001**** |
| High education, N, no degree/student/vocational training/university degree | 8/4/89/107 | 3/0/15/3 | 1/1/15/7 | 17.44 | 6 | **0.01*** |
| Monthly income^a^, M (SD) | 2019 (921) | 1412 (582) | 1841 (1001) | 7.69 | 2 | **0.021*** |
| **Physical Health** |  |  |  |  |  |  |
| BMI | 25.87 (6) | 28.31 (6.84) | 26.65 (5.08) | 1.67 | 2, 251 | 0.19 |
| Chronic illness, yes/no | 56/154 | 9/12 | 3/21 | 5.28 | 2 | 0.07 |
| Hospital admission (last year), yes/no | 56/154 | 12/9 | 18/6 | 8.81 | 2 | **0.01*** |
| **Mental Health** |  |  |  |  |  |  |
| Life-time diagnosis, yes/no | 111/82 | 15/3 | 19/2 | 12.39 | 2 | **0.002**** |
| **Substance Use** |  |  |  |  |  |  |
| Smoking, N, yes/no | 60/148 | 12/9 | 9/15 | 7.38 | 2 | **0.025*** |
| Alcohol use^b^, M (SD) | 3.96 (3.62) | 7.35 (7.36) | 3.10 (1.73) | 7.28 | 2 | **0.026*** |
| Cannabis use, N, yes/no | 28/180 | 7/14 | 1/23 | 8.37 | 2 | **0.015*** |
| **Social Indicators** |  |  |  |  |  |  |
| Partnership status, N, yes/no | 165/45 | 13/8 | 7/17 | 3.41 | 2 | 0.18 |
| Living alone, N, yes/no | 42/169 | 6/15 | 7/17 | 1.78 | 2 | 0.41 |
| Number of friends, N, none/1/2-3/4 and more | 3/9/84/114 | 2/2/13/4 | 0/4/6/14 | 21.65 | 6 | **0.001**** |

| Monthly friendship contact, N,  less than 1/1-2 times/3-4 times/5 and more | 8/18/40/144 | 0/3/6/11 | 1/3/6/13 | 4.24 | 6 | 0.64 |
| --- | --- | --- | --- | --- | --- | --- |
| Friendship quality, M (SD) | 2.96 (0.69) | 2.75 (0.64) | 2.74 (0.69) | 1.73 | 2, 250 | 0.18 |

^a^ adjusted income per person according to the organization of economic co-operation and development [household income/(adult number *0.5+ child number*0.3+1)]

^b^ Alcohol Use Disorders Identification Test

**Table S17.** Post-hoc tests for group comparisons: externalizing symptom trajectories.

|  | **Low vs. Increasing Symptoms** | | **Low vs. Decreasing Symptoms** | | **Increasing vs. Decreasing Symptoms** | |
| --- | --- | --- | --- | --- | --- | --- |
|  | Test Statistics | p | Test Statistics | p | Test Statistics | p |
| Monthly income | 2.69 | **0.021*** | 0.92 | 1 | -1.43 | 0.46 |
| Alcohol use | -0.58 | 1 | -2.57 | **0.03*** | -2.36 | 0.055 |

**Table S18.** Comparisons of internalizing symptom trajectories in terms of adult functioning.

|  | **Low Symptoms** | **Increasing Symptoms** | **Decreasing Symptoms** | **Test Statistics** | **df** | **p** |
| --- | --- | --- | --- | --- | --- | --- |
| **Demographics** |  |  |  |  |  |  |
| Sex, N, F/M | 111/93 | 22/12 | 11/7 | 1.41 | 2 | 0.49 |
| School degree, N, no degree/ secondary school/ intermediate school/ vocational school /high school | 1/28/55/14/106 | 0/1/17/4/12 | 0/4/7/1/6 | 13.28 | 8 | 0.10 |
| High education, N, no degree/student/vocational training/university degree | 8/3/87/103 | 2/2/22/8 | 2/0/10/6 | 13.76 | 6 | **0.032*** |
| Monthly income^a^, M(SD) | 2027 (950) | 1607 (697) | 1810 (805) | 3.22 | 2, 241 | **0.042*** |
| **Physical Health** |  |  |  |  |  |  |
| BMI | 25.97(5.78) | 27.78 (7.66) | 25.06 (4.56) | 1.64 | 2, 251 | 0.20 |
| Chronic illness, yes/no | 48/155 | 13/21 | 7/11 | 4.65 | 2 | 0.10 |
| Hospital admission (last year), yes/no | 50/153 | 19/15 | 5/13 | 13.82 | 2 | **0.001**** |
| **Mental Health** |  |  |  |  |  |  |
| Life-time diagnosis, yes/no | 103/80 | 25/6 | 17/1 | 15.21 | 2 | **0.000***** |
| **Substance Use** |  |  |  |  |  |  |
| Smoking, N, yes/no | 69/132 | 9/25 | 3/15 | 2.92 | 2 | 0.23 |
| Alcohol use^b^, M (SD) | 4.19 (4.22) | 4.55 (3.87) | 3.27 (2.25) | 0.50 | 2, 225 | 0.61 |
| Cannabis use, N, yes/no | 32/169 | 4/30 | 0/18 | 3.63 | 2 | 0.16 |
| **Social Indicators** |  |  |  |  |  |  |
| Partnership status, N, yes/no | 156/47 | 27/7 | 12/6 | 1.14 | 2 | 0.56 |
| Living alone, N, yes/no | 36/168 | 10/24 | 9/9 | 11.72 | 2 | **0.003**** |
| Number of friends, N, none/1/2-3/4 and more | 4/11/73/115 | 1/2/22/9 | 0/2/8/8 | 12.79 | 6 | **0.046*** |

| Monthly friendship contact, N, less than 1/1-2 times/3-4 times/ 5 and more | 7/17/39/138 | 2/4/7/21 | 0/3/6/9 | 5.10 | 6 | 0.53 |
| --- | --- | --- | --- | --- | --- | --- |
| Friendship quality, M (SD) | 2.99 (0.69) | 2.56 (0.71) | 2.89 (0.76) | 9.76 | 2 | **0.01**** |

^a^ adjusted income per person according to the organization of economic co-operation and development [household income/(adult number *0.5+ child number*0.3+1)]

^b^ Alcohol Use Disorders Identification Test

**Table S19.** Post-hoc tests for group comparisons: internalizing symptom trajectories.

|  | **Low vs. Increasing Symptoms** | | **Low vs. Decreasing Symptoms** | | **Increasing vs. Decreasing Symptoms** | |
| --- | --- | --- | --- | --- | --- | --- |
|  | Test Statistics | p | Test Statistics | p | Test Statistics | p |
| Montly income^a^ | 419.48 | **0.04*** | 216.96 | 0.62 | -202.52 | 0.74 |
|  |  |  |  |  |  |  |
| Friendship quality^b^ | 38.03 | **0.006**** | 9.90 | 1 | -28.17 | 0.43 |

a One-way ANOVA + Tukey-HSD test

b Kruskal-Wallis test

# Clinical and Psychosocial Correlates of Symptom Change

# Treatment

**General Overview**

**T2-T3:** Thirty-one participants received a treatment due to their behavioral problems between T2 (age 2) and T3 (age 4.5). Fourteen of them received consultation, three of them received ergotherapy, one of them received speech therapy, and 13 of them received other treatments.

**T3-T4:** Seventy-eight participants received treatment due to their behavioral problems between T3 (age 4.5) and T4 (age 8): Consultation (n=24), ergotherapy (n=10), play therapy (n=7), behavioral therapy (n=8), medication (n=1), speech therapy (n=1), family help (n=1), and other treatments (n=25).

**T4-T5:** Seventy participants received treatment because of their behavioral problems between T4 (age 8) and T5 (age 11): Consultation (n=21), ergotherapy (n=2), play therapy (n=16), behavioral therapy (n=9), medication (n=3), speech therapy (n=6), and other treatments (n=13).

**T5-T6:** Forty-nine participants received treatment because of their behavioral problems between T5 (age 11) and T6 (age 15): Consultation (n=12), ergotherapy (n=3), play therapy (n=11), behavioral therapy (n=10), medication (n=5), speech therapy (n=1), family help (n=1), and other treatments (n=6).

**T6-T7:** Fifty-five participants received treatment because of their behavioral problems between T6 (age 15) and T7 (age 19): Consultation (n=19), person-centered psychotherapy (n=20), behavioral therapy (n=11), medication (n=4), and other treatments (n=1).

From T8 (age 22) onwards, participants self-reported their medication and/or psychotherapy use in the last year.

**T8 (age 22):** Twenty participants used medications, and 23 participants received psychotherapy.

**T9 (age 23):** Fourteen participants used medications, and 19 participants received psychotherapy.

**T10 (age 25):** Eighteen participants used medications, and 18 participants received psychotherapy.

**T11 (age 33):** Twenty-eight participants used medications, and 44 participants received psychotherapy.

# Group Differences

**Table S20.** Treatment use for psychological problems: externalizing symptom trajectories

|  | **Time** | **Low Symptoms** | **Increasing Symptoms** | **Decreasing Symptoms** | **χ2** | **df** | **p** |
| --- | --- | --- | --- | --- | --- | --- | --- |
| Treatment, yes/no | T3 | 24/231 | 2/25 | 5/30 | 1.02 | 2 | 0.60 |
| Treatment, yes/no | T4 | 55/197 | 7/20 | **16/19** | 9.41 | 2 | **0.009**** |
| Treatment, yes/no | T5 | 47/207 | 5/22 | **18/17** | 19.56 | 2 | **0.000***** |
| Treatment, yes/no | T6 | 35/218 | 6/20 | 8/27 | 3.10 | 2 | 0.21 |
| Treatment, yes/no | T7 | 39/216 | **10/17** | 6/29 | 8.05 | 2 | **0.018** |
| Medication, yes/no | T8 | 14/239 | 3/24 | 3/32 | 1.60 | 2 | 0.45 |
| Psychotherapy, yes/no | T8 | 18/235 | 1/26 | 4/31 | 1.41 | 2 | 0.49 |
| Medication, yes/no | T9 | 10/242 | 2/25 | 2/31 | 0.89 | 2 | 0.64 |
| Psychotherapy, yes/no | T9 | 15/237 | 2/25 | 2/31 | 0.09 | 2 | 0.96 |
| Medication, yes/no | T10 | 16/235 | 2/23 | 0/33 | 2.40 | 2 | 0.30 |
| Psychotherapy, yes/no | T10 | 8/243 | 3/22 | 1/32 | 4.80 | 2 | 0.09 |
| Medication, yes/no | T11 | 19/184 | **6/15** | 3/21 | 7.05 | 2 | **0.029*** |
| Psychotherapy, yes/no | T11 | 37/170 | 3/18 | 4/20 | 0.18 | 2 | 0.91 |

*p < 0.05; * p < 0.01; *** p< 0.001

**Table S21.** Treatment use for psychological problems: internalizing symptom trajectories

|  | **Time** | **Low Symptoms** | **Increasing Symptoms** | **Decreasing Symptoms** | **χ2** | **df** | **p** |
| --- | --- | --- | --- | --- | --- | --- | --- |
| Treatment, yes/no | T3 | 23/229 | 4/39 | 4/18 | 1.89 | 2 | 0.39 |
| Treatment, yes/no | T4 | 54/195 | 12/31 | **12/10** | 11.94 | 2 | **0.003**** |
| Treatment, yes/no | T5 | 53/198 | 8/35 | 9/13 | 4.96 | 2 | 0.08 |
| Treatment, yes/no | T6 | 34/215 | 11/32 | 4/18 | 4.08 | 2 | 0.13 |
| Treatment, yes/no | T7 | 28/224 | **20/23** | 7/15 | 35.55 | 2 | **0.000***** |
| Medication, yes/no | T8 | 13/237 | 6/37 | 1/21 | 4.86 | 2 | 0.09 |
| Psychotherapy, yes/no | T8 | 14/236 | **7/36** | 2/20 | 6.29 | 2 | **0.043*** |
| Medication, yes/no | T9 | 4/243 | **8/35** | 2/20 | 28.82 | 2 | **0.000***** |
| Psychotherapy, yes/no | T9 | 8/239 | **8/35** | 3/19 | 17.48 | 2 | **0.000***** |
| Medication, yes/no | T10 | 12/234 | 5/36 | 1/21 | 3.5 | 2 | 0.17 |
| Psychotherapy, yes/no | T10 | 6/240 | **4/37** | 2/20 | 6.76 | 2 | **0.034*** |
| Medication, yes/no | T11 | 17/182 | **9/24** | 2/14 | 9.94 | 2 | **0.007**** |
| Psychotherapy, yes/no | T11 | 26/175 | **16/18** | 2/15 | 23.91 | 2 | **0.000***** |

*p < 0.05; * p < 0.01; *** p< 0.001

# Stressful Life Events

**Table S22.** Comparisons of externalizing symptoms trajectories in terms of stress life events.

|  | **Low Symptoms** | **Increasing Symptoms** | **Decreasing Symptoms** | **Test Statistics** | **df** | **p** |
| --- | --- | --- | --- | --- | --- | --- |
| Life events T1, Mean (SD) | 3.68 (2.27) | 4.89 (3.31) | 4.23 (2.09) | 3.75 | 2, 314 | **0.024*** |
| Life events T2, Median (IQR) | 5 (4) | 6 (4) | 5 (4) | 7.87 | 2 | **0.02*** |
| Life events T3, Mean (SD) | 6.78 (3.41) | 7.59 (3.32) | 7.83 (4.10) | 1.87 | 2,314 | 0.16 |
| Life events T4, Median (IQR) | 6 (4) | 6 (4) | 7 (5) | 9.73 | 2 | **0.008**** |
| Life events T5, Median (IQR) | 4 (4) | 6 (5) | 7 (5) | 15.99 | 2 | **0.000***** |
| Life events T6, Median (IQR) | 5 (4) | 6 (5) | 7 (5) | 6.73 | 2 | **0.035*** |
| Life events T7, Median (IQR) | 6 (5) | 9 (11) | 9 (8) | 20.25 | 2 | **0.000***** |
| Life events T8, Median (IQR) | 6 (5) | 10 (5) | 8 (5) | 18.40 | 2 | **0.000***** |
| Life events T9, Median (IQR) | 3 (3) | 5 (5) | 4 (3) | 19.73 | 2 | **0.000***** |
| Life events T10, Median (IQR) | 4 (4) | 8 (7) | 3 (4) | 14.10 | 2 | **0.001**** |
| Life events T11, Median (IQR) | 3 (4) | 8 (5) | 3 (3) | 14.26 | 2 | **0.001**** |

* p < 0.05; ** p < 0.01; *** p < 0.001

53

**Table S23.** Post-hoc tests for group comparisons: externalizing symptom trajectories.

|  | **Low vs. Increasing Symptoms** | | **Low vs. Decreasing Symptoms** | | **Increasing vs.**  **Decreasing Symptoms** | |
| --- | --- | --- | --- | --- | --- | --- |
|  | Test Statistics | p | Test Statistics | p | Test Statistics | p |
| Life events T1^a^ | -1.21 | **0.031*** | -0.55 | 0.40 | 0.66 | 0.52 |
| Life events T2^b^ | -44.23 | **0.048*** | -27.67 | 0.27 | 16.56 | 1 |
| Life events T4^b^ | -18.63 | 0.93 | -49.85 | **0.007**** | -31.22 | 0.54 |
| Life events T5^b^ | -46.64 | **0.034*** | -54.88 | **0.002**** | -8.24 | 1 |
| Life events T6^b^ | -10.25 | 1 | -42.19 | **0.01*** | -31.94 | 0.52 |
| Life events T7^b^ | -53.2 | **0.012*** | -61.7 | **0.001**** | -8.5 | 1 |
| Life events T8^b^ | -70.66 | **0.000***** | -37.62 | 0.064 | 33.04 | 0.47 |
| Life events T9^b^ | -78.70 | **0.000***** | -21.76 | 0.56 | 56.93 | **0.042*** |
| Life events T10^b^ | -67.15 | **0.001**** | 11.22 | 1 | 78.34 | **0.003**** |
| Life events T11^b^ | -60.65 | **0.001**** | 7.65 | 1 | 68.30 | **0.007**** |

^a^ One-way ANOVA+ Tukey Test; ^b^ Kruskal-Wallis test; * p < 0.05; ** p < 0.01; *** p < 0.001 Notes: All p values were corrected with Bonferroni correction.

54

**Table S24.** Comparisons of internalizing symptom trajectories in terms of stress life events.

|  | **Low Symptoms** | **Increasing Symptoms** | **Decreasing Symptoms** | **Test Statistics** | **df** | **p** |
| --- | --- | --- | --- | --- | --- | --- |
| Life events T1, Median, (IQR) | 3 (3) | 5 (4) | 4 (3) | 6.10 | 2 | 0.05 |
| Life events T2, Median, (IQR) | 5 (4) | 7 (5) | 5.5 (4) | 11.01 | 2 | **0.004**** |
| Life events T3, Median, (IQR) | 6 (5) | 6 (3) | 8 (6) | 9.39 | 2 | **0.009**** |
| Life events T4, Mean (SD) | 6.57 (3.41) | 6.67 (3.70) | 8.18 (5.18) | 2.05 | 2,313 | 0.13 |
| Life events T5, Median, (IQR) | 5 (4) | 5 (4) | 8.5 (6) | 9.13 | 2 | **0.01*** |
| Life events T6, Mean (SD) | 6.08 (3.53) | 6.22 (4.17) | 7.25 (2.53) | 1 | 2 | 0.37 |
| Life events T7, Median, (IQR) | 6 (5) | 9 (7) | 7.5 (8) | 11.77 | 2 | **0.003**** |
| Life events T8, Median, (IQR) | 6 (5) | 9 (4) | 7.5 (7) | 16.27 | 2 | **0.000***** |
| Life events T9, Median, (IQR) | 3 (3) | 5 (3) | 4 (3) | 18.19 | 2 | **0.000***** |
| Life events T10, Median, (IQR) | 3 (4) | 6 (6) | 4 (5) | 21.21 | 2 | **0.000***** |
| Life events T11, Median, (IQR) | 3 (3) | 5 (6) | 3 (4) | 17.34 | 2 | **0.000***** |

* p < 0.05; ** p < 0.01; *** p < 0.001

55

**Table S25.** Post-hoc tests for group comparisons: internalizing symptom trajectories.

|  | **Low vs. Increasing Symptoms** | | **Low vs. Decreasing Symptoms** | | **Increasing vs. Decreasing Symptoms** | |
| --- | --- | --- | --- | --- | --- | --- |
|  | Test Statistics | p | Test Statistics | p | Test Statistics | p |
| Life events T2^a^ | -49.41 | **0.003**** | -14.51 | 1 | 34.90 | 0.43 |
| Life events T3^a^ | -21.34 | 0.47 | -57.82 | **0.013*** | -36.48 | 0.38 |
| Life events T5^a^ | -5.53 | 1 | -61.05 | **0.008**** | -55.52 | 0.06 |
| Life events T7^a^ | -43.94 | **0.011*** | -42.89 | 0.10 | 1.04 | 1 |
| Life events T8^a^ | -59.25 | **0.000***** | -24.41 | 0.68 | 34.84 | 0.43 |
| Life events T9^a^ | -60.5 | **0.000***** | -31.80 | 0.33 | 28.7 | 0.66 |
| Life events T10^a^ | -68.63 | **0.000***** | -19.54 | 0.98 | 49.09 | 0.11 |
| Life events T11^a^ | -54.23 | **0.000***** | -5.66 | 1 | 48.57 | 0.06 |

^a^ Kruskal-Wallis test; * p < 0.05; ** p < 0.01; *** p < 0.001 Notes: All p values were corrected with Bonferroni correction.

# Coping

**Table S26.** Comparisons of externalizing symptom trajectories in terms of coping ratings.

|  | **Low Symptoms** | **Increasing Symptoms** | **Decreasing Symptoms** | **Test Statistics** | **df** | **p** |
| --- | --- | --- | --- | --- | --- | --- |
| Stress coping T4, Median (IQR) | 4 (1) | 4 (1) | 4 (2) | 6.63 | 2 | **0.036*** |
| Daily coping T4, Mean (SD) | 4.55 (0.69) | 4.44 (0.75) | 4.23 (0.77) | 3.24 | 2 | **0.041*** |
| Stress coping T5, Median (IQR) | 4 (1) | 4 (1) | 4 (2) | 7.29 | 2 | **0.03*** |
| Daily coping T5, Median (IQR) | 5 (1) | 5 (1) | 4 (1) | 9.43 | 2 | **0.009**** |

**Table S27.** Post-hoc group comparisons: externalizing symptom trajectories.

|  | **Low vs. Increasing Symptoms** | | **Low vs. Decreasing Symptoms** | | **Increasing vs.**  **Decreasing Symptoms** | |
| --- | --- | --- | --- | --- | --- | --- |
|  | Test Statistics | p | Test Statistics | p | Test Statistics | p |
| Stress coping T4^a^ | 27.31 | 0.34 | 33.87 | 0.08 | 6.56 | 1 |
| Daily coping T4^b^ | 0.10 | 0.75 | 0.32 | **0.034*** | 0.22 | 0.46 |
| Stress coping T5^a^ | 13.46 | 1 | 40.89 | **0.02*** | 27.44 | 0.63 |
| Daily coping T5^a^ | -3.44 | 1 | 44.01 | **0.008*** | 47.45 | 0.065 |

^a^ Kruskal-Wallis Test ^b^ One-way ANOVA + Tukey test; * p < 0.05; ** p < 0.01; *** p < 0.001 Notes: All p values were corrected with Bonferroni correction.

**Table S28.** Comparisons of internalizing symptom trajectories in terms of coping ratings.

|  | **Low Symptoms** | **Increasing Symptoms** | **Decreasing Symptoms** | **Test Statistics** | **df** | **p** |
| --- | --- | --- | --- | --- | --- | --- |
| Stress coping T4, Median (IQR) | 4 (1) | 4 (1) | 3 (2) | 11.68 | 2 | **0.003**** |
| Daily coping T4, Median (IQR) | 5 (1) | 4 (1) | 4 (1) | 14.79 | 2 | **0.001**** |
| Stress coping T5, Median (IQR) | 4 (1) | 4 (1) | 3.5 (1) | 12.29 | 2 | **0.002**** |
| Daily coping T5, Median (IQR) | 5 (1) | 5 (1) | 4 (1) | 10.68 | 2 | **0.005**** |

**Table S29.** Post-hoc tests for group comparisons: internalizing symptom trajectories.

|  | **Low vs. Increasing Symptoms** | | **Low vs. Decreasing Symptoms** | | **Increasing vs.**  **Decreasing Symptoms** | |
| --- | --- | --- | --- | --- | --- | --- |
|  | Test Statistics | p | Test Statistics | p | Test Statistics | p |
| Stress coping T4^a^ | -0.53 | 1 | 64.52 | **0.002**** | 65.05 | **0.11*** |
| Daily coping T4^a^ | 16.90 | 0.63 | 68.28 | **0.001**** | 51.38 | 0.05 |
| Stress coping T5^a^ | 5.13 | 1 | 66.63 | **0.001**** | 61.51 | **0.02*** |
| Daily coping T5^a^ | 14.31 | 0.85 | 57.18 | **0.004**** | 42.87 | 0.13 |

^a^ Kruskal-Wallis Test; * p < 0.05; ** p < 0.01; *** p < 0.001 Notes: All p values were corrected with Bonferroni correction.

**Table S30.** Comparisons of externalizing symptom trajectories in terms of stress coping styles in adulthood (T8).

|  | **Low Symptoms** | **Increasing Symptoms** | **Decreasing Symptoms** | **Test Statistics** | **df** | **p** |
| --- | --- | --- | --- | --- | --- | --- |
| Devaluation/Defense, Mean (SD) | 11.25 (3.88) | 10.65 (3.68) | 9.65 (3.55) | 2.66 | 2 | 0.07 |
| Distraction, Median (IQR) | 11.5 (4.5) | 11.5 (3.5) | 10 (5) | 6.50 | 2 | **0.039*** |
| Control, Median (IQR) | 15.67 (4.67) | 13.67 (2.67) | 14.67 (5) | 12.29 | 2 | **0.002**** |
| Negative strategies, Median (IQR) | 8.5 (5.25) | 11 (7) | 7.75 (6.5) | 7.08 | 2 | **0.029*** |

**Table S31.** Post-hoc tests for group comparisons: Externalizing symptom trajectories.

|  | **Low vs. Increasing Symptoms** | | **Low vs. Decreasing Symptoms** | | **Increasing vs.**  **Decreasing Symptoms** | |
| --- | --- | --- | --- | --- | --- | --- |
|  | Test Statistics | p | Test Statistics | p | Test Statistics | p |
| Distraction^a^ | -6.96 | 1 | -40.74 | **0.041*** | -47.70 | 0.19 |
| Control^a^ | 58.20 | **0.004**** | 28.63 | 0.25 | -29.57 | 0.61 |
| Negative Strategies^a^ | -48.16 | **0.023*** | -4.19 | 1 | 43.97 | 0.17 |

^a^ Kruskal-Wallis Test; * p < 0.05; ** p < 0.01; *** p < 0.001 Notes: All p values were corrected with Bonferroni correction.

**Table S32.** Comparisons of internalizing symptom trajectories in terms of stress coping styles in adulthood (T8).

|  | **Low Symptoms** | **Increasing Symptoms** | **Decreasing Symptoms** | **Test Statistics** | **df** | **P** |
| --- | --- | --- | --- | --- | --- | --- |
| Devaluation, Median (IQR) | 12 (5) | 8 (5.5) | 8.75 (6.13) | 18.55 | 2 | **0.000***** |
| Distraction, Mean (SD) | 11.14 (3.8) | 11.21 (3.52) | 9.73 (3.8) | 1.46 | 2 | 0.23 |
| Control, Median (IQR) | 15.83 (4.33) | 13.67 (4) | 14.67 (4.67) | 9.85 | 2 | **0.007**** |
| Negative strategies, Median (IQR) | 8.25 (4.44) | 13.75 (4.5) | 10.5 (5.31) | 55.18 | 2 | **0.000***** |

**Table S33.** Post-hoc tests for group comparisons: internalizing symptom trajectories.

|  | **Low vs. Increasing Symptoms** | | **Low vs. Decreasing Symptoms** | | **Increasing vs. Decreasing Symptoms** | |
| --- | --- | --- | --- | --- | --- | --- |
|  | Test Statistics | p | Test Statistics | p | Test Statistics | p |
| Devaluation^a^ | 53.58 | **0.001**** | 53.81 | **0.02*** | -0.23 | 1 |
| Control^a^ | 43.74 | **0.009**** | 27.11 | 0.52 | -16.63 | 1 |
| Negative Strategies^a^ | -108.59 | **0.000***** | -37.53 | 0.17 | 71.05 | **0.001**** |

^a^ Kruskal-Wallis Test; * p < 0.05; ** p < 0.01; *** p < 0.001 Notes: All p values were corrected with Bonferroni correction.

# Resources Social Support

**Table S34.** Comparisons of externalizing symptom trajectories in terms of social support.

|  | **Low Symptoms** | **Increasing Symptoms** | **Decreasing Symptoms** | **Test Statistics** | **df** | **p** |
| --- | --- | --- | --- | --- | --- | --- |
| Social support T4, M (SD) | 4.82 (0.7) | 4.59 (0.84) | 4.46 (0.89) | 4.47 | 2 | **0.02*** |
| Social integration T4, Median (IQR) | 5 (1) | 5 (1) | 5 (2) | 7.07 | 2 | **0.03*** |
| Social support T5, Median (IQR) | 5 (1) | 5 (1) | 4 (1) | 7.92 | 2 | **0.02*** |
| Social integration T5, Median (IQR) | 5 (1) | 5 (1) | 4 (1) | 11.03 | 2 | **0.004**** |

**Table S35.** Post-hoc tests for group comparisons: externalizing symptom trajectories.

|  | **Low vs. Increasing Symptoms** | | **Low vs. Decreasing Symptoms** | | **Increasing vs.**  **Decreasing Symptoms** | |
| --- | --- | --- | --- | --- | --- | --- |
|  | Test Statistics | p | Test Statistics | p | Test Statistics | p |
| Social support T4^a^ | 0.23 | 0.29 | 0.36 | **0.018*** | 0.14 | 0.75 |
| Social integration T4^b^ | 12.44 | 1 | 39.88 | **0.009**** | 27.44 | 0.61 |
| Social support T5^b^ | 7.45 | 1 | 49.95 | **0.003**** | 41.60 | 0.13 |
| Social integration T5^b^ | 14.15 | 1 | 41.89 | **0.017*** | 27.75 | 0.59 |

^a^ One-way ANOVA + Tukey test ^b^ Kruskal-Wallis Test; * p < 0.05; ** p < 0.01; *** p < 0.001 Notes: All p values were corrected with Bonferroni correction.

**Table S36.** Comparisons of internalizing symptom trajectories in terms of social support.

|  | **Low Symptoms** | **Increasing Symptoms** | **Decreasing Symptoms** | **Test Statistics** | **df** | **p** |
| --- | --- | --- | --- | --- | --- | --- |
| Social support T4, Median (IQR) | 5 (1) | 5 (1) | 4 (1) | 12.51 | 2 | **0.002**** |
| Social integration T4, Median (IQR) | 5 (1) | 5 (1) | 4 (2) | 9.77 | 2 | **0.008**** |
| Social support T5, Median (IQR) | 5 (1) | 5 (1) | 4 (1) | 16.62 | 2 | **0.000***** |
| Social integration T5, Median (IQR) | 5 (1) | 5 (1) | 4 (1) | 14.73 | 2 | **0.001**** |

**Table S37.** Post-hoc tests for group comparisons: internalizing symptom trajectories.

|  | **Low vs. Increasing Symptoms** | | **Low vs. Decreasing Symptoms** | | **Increasing vs.**  **Decreasing Symptoms** | |
| --- | --- | --- | --- | --- | --- | --- |
|  | Test Statistics | p | Test Statistics | p | Test Statistics | p |
| Social support T4^a^ | 24.16 | 0.20 | 56.96 | **0.004**** | 32.80 | 0.35 |
| Social integration T4^a^ | 14.38 | 0.91 | 57.16 | **0.007**** | 42.78 | 0.16 |
| Social support T5^a^ | 18.40 | 0.51 | 72.79 | **0.000***** | 54.39 | 0.13 |
| Social integration T5^a^ | 11.63 | 1 | 71.32 | **0.000***** | 59.69 | **0.02*** |

^a^ Kruskal-Wallis Test. * p < 0.05; ** p < 0.01; *** p < 0.001 Notes: All p values were corrected with Bonferroni correction.

**Table S38.** Comparisons of externalizing symptom trajectories in terms of resources in young-adulthood.

|  | **Low Symptoms** | **Increasing Symptoms** | **Decreasing Symptoms** | **Test Statistics** | **df** | **p** |
| --- | --- | --- | --- | --- | --- | --- |
| Partner, Mean (SD) | 7.27 (4.83) | 7.75 (3.92) | 8.73 (4.02) | 1.46 | 2 | 0.23 |
| Friends and family, Median (IQR) | 8 (2) | 7 (2) | 8 (3) | 10.69 | 2 | **0.005**** |
| Work, Mean (SD) | 7.49 (3.05) | 6.58 (2.87) | 7.76 (3.26) | 1.15 | 2 | 0.32 |
| Leisure, Median (IQR) | 5 (3) | 3 (3) | 5 (2) | 6.28 | 2 | **0.043*** |
| Home, Mean (SD) | 4.54 (1.48) | 3.96 (1.60) | 4.76 (1.32) | 2.19 | 2 | 0.11 |
| Religion, Mean (SD) | 3.80 (2.43) | 3.13 (2.40) | 3.15 (2.11) | 1.79 | 2 | 0.17 |

**Table S39.** Post-hoc tests for group comparisons: externalizing symptom trajectories.

|  | **Low vs. Increasing Symptoms** | | **Low vs. Decreasing Symptoms** | | **Increasing vs.**  **Decreasing Symptoms** | |
| --- | --- | --- | --- | --- | --- | --- |
|  | Test Statistics | p | Test Statistics | p | Test Statistics | p |
| Friends and family^a^ | 56.07 | **0.006**** | 22.27 | 0.48 | -33.80 | 0.42 |
| Leisure^a^ | 44.56 | 0.053 | 9.31 | 1 | -53.87 | 0.068 |

^a^ Kruskal-Wallis Test * p < 0.05; ** p < 0.01; *** p < 0.001 Notes: All p values were corrected with Bonferroni correction.

**Table S40.** Comparisons of internalizing symptom trajectories in terms of resources in young-adulthood.

|  | **Low Symptoms** | **Increasing Symptoms** | **Decreasing Symptoms** | **Test Statistics** | **df** | **p** |
| --- | --- | --- | --- | --- | --- | --- |
| Partner, Mean (SD) | 7.63 (4.69) | 6.12 (4.73) | 8.14 (4.23) | 2.07 | 2 | 0.13 |
| Friends and family, Median (IQR) | 8 (2) | 7 (2) | 8 (2) | 16.97 | 2 | **0.000***** |
| Work, Mean (SD) | 7.61 (3.01) | 6.68 (3.16) | 7 (3.29) | 1.86 | 2 | 0.16 |
| Leisure, Median (IQR) | 5 (3) | 3 (3) | 4 (2) | 11.72 | 2 | **0.003**** |
| Home, Median (IQR) | 5 (2) | 4 (3) | 5 (2) | 17.47 | 2 | **0.000***** |
| Religion, Median (IQR) | 3 (3) | 2 (4) | 4 (4) | 6.12 | 2 | **0.047*** |

**Table S41.** Post-hoc tests for group comparisons: internalizing symptom trajectories.

|  | **Low vs. Increasing Symptoms** | | **Low vs. Decreasing Symptoms** | | **Increasing vs.**  **Decreasing Symptoms** | |
| --- | --- | --- | --- | --- | --- | --- |
|  | Test Statistics | p | Test Statistics | p | Test Statistics | p |
| Friends and family^a^ | 57.81 | **0.000***** | 25.66 | 0.56 | -32.15 | 0.48 |
| Leisure^a^ | 50.51 | **0.002***** | 14.75 | 1 | -35.76 | 0.39 |
| Home^a^ | 59.70 | **0.000***** | 6.19 | 1 | -65.89 | **0.013*** |
| Religion^a^ | 32.22 | 0.09 | 19.05 | 1 | -51.28 | 0.09 |

^a^ Kruskal-Wallis Test * p < 0.05; ** p < 0.01; *** p < 0.001 Notes: All p values were corrected with Bonferroni correction.

# Appendix S9. Sensitivity Analyses

# Trajectory Modeling with Full Available Data

To ensure robust estimation of psychopathology symptom trajectories, the primary analyses included participants with data available for most assessment waves (≥5 of 8; n = 317, 171 females). As a sensitivity analysis, we repeated the same modeling procedure including all participants with at least three available assessments (n = 342, 180 females).

The three-class solutions for both externalizing and internalizing symptoms were replicated (Tables S42–S43). Growth trajectories (Figure S10) and class distributions (Tables S42–S43) were highly consistent with the primary analyses. Class membership demonstrated excellent stability (0.98 for externalizing and 0.99 for internalizing symptom classes), indicating a high degree of concordance with the original class assignments.

**Figure S10.** Identified class solution for externalizing (A) and internalizing (B) symptoms trajectories. Purple, green, and yellow colors represent low, increasing, and decreasing symptoms classes, respectively.

**Table S42.** Model fit indices of latent growth mixture models for externalizing symptoms (n=342).

| Class | AIC | BIC | Entropy | Class Membership, N(%) | LRT | Adjuste d LRT |
| --- | --- | --- | --- | --- | --- | --- |
| 1-class | -5563 | -5514 | - | - | - | - |
| 2-class | -5595 | -5542 | 0.89 | 31 (9%), 311 (91%) | 0.001 | 0.002 |
| **3-class** | **-5665** | **-5600** | **0.86** | **280 (82%), 34 (10%), 28 (8%)** | **0.019** | **0.022** |
| 4-class | -5688 | -5611 | 0.85 | 34(10%), 13(4%), 266(78%),  29(8%) | 0.18 | 0.19 |
| 5-class | -5705 | -5617 | 0.86 | 14(4%), 264(77%), 30(9%),  32(11%), 2(<1%) | 0.029 | 0.032 |

Abbreviations: AIC, Akaike Information Criterion; BIC, Bayesian Information Criterion; LRT, likelihood ratio test.

**Table S43.** Model fit indices of latent growth mixture models for internalizing symptoms (n=342).

| Class | AIC | BIC | Entropy | Class Membership, N(%) | LRT | Adjuste d LRT |
| --- | --- | --- | --- | --- | --- | --- |
| 1-class | -5128 | -5078 | - | - | - | - |
| 2-class | -5203 | -5141 | 0.86 | 289 (85%), 53 (15%) | 0.012 | 0.014 |
| **3-class** | **-5258** | **-5185** | **0.87** | **25 (7%), 43 (13%), 274 (80%)** | **0.023** | **0.028** |
| 4-class | -5275 | -5191 | 0.87 | 24(7%), 260(76%), 47(14%), 11  (3%) | 0.30 | 0.32 |
| 5-class | -5269 | -5181 | 0.74 | 61(18%), 47(14%), 11(3%),  19(5%), 204 (60%) | 0.39 | 0.41 |

Abbreviations: AIC, Akaike Information Criterion; BIC, Bayesian Information Criterion; LRT, likelihood ratio test.

# Permutation Tests

To assess the robustness and statistical significance of the machine-learning predictions, we conducted permutation tests to determine whether model performance exceeded chance level. To limit computational burden, 100 permutation iterations were conducted only for the best-performing models identified in the primary analyses. In each iteration, outcome labels were randomly permuted and models were refitted using nested cross-validation (5-fold inner CV; 10-fold outer CV) to generate null distributions for macro and class-specific AUC. Observed performance was compared against these null distributions to account for chance performance.

For externalizing symptom trajectories, the observed macro AUC (M = 0.77, SD = 0.07) was significantly higher than the macro AUC derived from the permutation-based null distribution (M = 0.49, SD = 0.04; p < 0.01). Similarly, all observed class-specific AUCs (low symptoms: M = 0.74, SD = 0.10; increasing symptoms: M = 0.77, SD = 0.07; decreasing symptoms: M = 0.78, SD = 0.13) were significantly greater than the corresponding class-specific AUCs from the null distributions (low symptoms: M = 0.49, SD = 0.05; increasing symptoms: M = 0.49, SD = 0.07; decreasing symptoms: M = 0.49, SD = 0.06; all p < 0.01).

For internalizing symptom trajectories, the observed macro AUC (M = 0.75, SD = 0.09) was significantly higher than the macro AUC derived from the permutation-based null distribution (M = 0.51, SD = 0.04; p < 0.01). Similarly, all observed class-specific AUCs (low symptoms: M = 0.73, SD = 0.11; increasing symptoms: M = 0.71, SD = 0.13; decreasing symptoms: M = 0.80, SD = 0.17) were significantly greater than the corresponding class-specific AUCs from the null distributions (low symptoms: M = 0.51, SD = 0.05; increasing symptoms: M = 0.50, SD = 0.06; decreasing symptoms: M = 0.52, SD = 0.08; all p < 0.01).

# Model Predictions with Childhood Features Only

We performed machine learning analyses with the same settings using the features collected in childhood only (0-8 years) (Table S44).

**Table S44.** Feature List including measures collected up to age 8.

| **Feature** | **Measurement Time** | **N** | **Range** | **Mean (SD) or n (%)** | **Brief Description** |
| --- | --- | --- | --- | --- | --- |
| Sex | T1 | 317 | 0-1 | 171 (53.9%)  female | Biological sex at birth (0=male, 1=female) |
| PGS ADHD | T6 | 298 | -0.0004 –  0.0004 | 0.0000  (0.0001) | Polygenic risk score for attention deficit and hyperactivity disorder |
| PGS AG | T6 | 298 | -0.0437-  0.0347 | 0.0001  (0.0135) | Polygenic risk score for aggression |
| PGS Alcohol | T6 | 298 | -0.0145 –  0.0146 | 0 (0.0049) | Polygenic risk score for alcohol dependence |
| PGS Anxiety | T6 | 298 | -0.0003 –  0.0004 | 0 (0.0001) | Polygenic risk score for anxiety |
| PGS MDD | T6 | 298 | -0.0001 –  0.0001 | 0 (0.00003) | Polygenic risk score for major depressive disorder |
| PGS SCZ | T6 | 298 | -0.0003 –  0.0004 | 0 (0.0001) | Polygenic risk score for schizophrenia |
| PGS CP | T6 | 298 | -0.0001 –  0.0001 | 0 (0.00002) | Polygenic score for cognitive performance |
| PGS EA | T6 | 298 | -0.00004-  0.00004 | 0 (0.00001) | Polygenic score for educational attainment |
| PGS IQ | T6 | 298 | -0.0001 –  0.0001 | 0 (0.00003) | Polygenic score for intelligent quotient |
| PGS NCEA | T6 | 298 | -0.0002 –  0.0002 | 0 (0.00006) | Polygenic score for non-cognitive educational attainment |
| Obstetric risk | T1 | 317 | 0-4 | 1 (1) | Birth complications, preterm birth, low birth weight |
| Moderate maternal smoking | T1 | 317 | 0-1 | 31 (9.8%) | Prenatal maternal smoking: Daily consumption up to 5 cigarettes |
| Heavy maternal smoking | T1 | 317 | 0-1 | 48 (15.1%) | Prenatal maternal smoking: Daily consumption more than 5 cigarettes |
| Maternal stress | T1 | 317 | 0-10 | 3 (2.1) | Sum of 11 items covering negative experiences during second or third trimester of pregnancy |
| Maternal psychosomatic complaints | T1 | 317 | 28-84 | 46.3 (10) | Mother’s psychological and somatic complaints in the last year |

| Income-to-need ratio | T1 | 317 | -0.8 – 0.8 | 0.2 (0.2) | Economical well-being of family compared to national average (Log-transformed scores) |
| --- | --- | --- | --- | --- | --- |
| Lower maternal stimulation | T1 | 304 | -8.2 – 7.2 | 0.02 (2.5) | Mothers’ attempts (vocal, facial or motor) to draw infants’ attention (video-recorded) [z-transformed reversely coded scores] |
| Low parental education | T1-T4 | 315 | 0-4 | 0.7 (1.4) | Parent without educational qualifications or skilled job training |
| Overcrowding | T1-T4 | 315 | 0-4 | 0.2 (0.6) | Less than one room per person in the home or home size < 50 m2 |
| Parental psychopathology | T1-T4 | 315 | 0-4 | 1.1 (1.3) | Moderate or severe disorder according to DSM-III-R criteria |
| Parental broken home | T1-T4 | 315 | 0-4 | 0.7 (1.4) | Institutional care of a parent/more than 2 changes of parental figures until the age of 18 or history of parental delinquency |
| Marital discord | T1-T4 | 315 | 0-4 | 0.7 (1.1) | Low quality of partnership in 2 out of 3 areas (harmony, communication, emotional warmth) |
| Unwanted pregnancy | T1 | 317 | 0-1 | 46 (14.5%) | An abortion is seriously considered |
| One parent family | T1-T4 | 315 | 0-4 | 0.5 (1.1) | One parent family / single parent |
| Early parenthood | T1 | 317 | 0-1 | 82 (25.9%) | Age of a parent ≤18 years at child’s birth or relationship between parents lasting <6 months at time of conception |
| Poor social integration | T1-T4 | 315 | 0-4 | 0.3 (0.7) | Lack of friends/of support in everyday life |
| Severe chronic events | T1-T4 | 315 | 0-4 | 1.2 (1.1) | Affecting a parent lasting >1 year |
| Poor coping skills - Parents | T1-T4 | 315 | 0-4 | 1.7 (1.5) | Inadequate coping with stressful events of the past year |
| Stressful life events | T1 | 317 | 0-15 | 3.8 (2.4) | Total stress exposure scores (prenatal-3 months) |
| Stressful life events | T2 | 316 | 0-21 | 5.4 (3) | Total stress exposure scores (3 months-2 years) |
| Stressful life events | T3 | 317 | 1-22 | 7 (3.5) | Total stress exposure scores (2 years-4.5 years) |
| Stressful life events | T4 | 316 | 0-23 | 6.9 (3.6) | Total stress exposure scores (4.5 years-8 years) |
| Behavioral symptoms | T1 | 317 | 0-11 | 2.1 (2.2) | Total behavioral symptoms at T1 |
| Behavioral symptoms | T2 | 316 | 0-19 | 4.4 (3.9) | Total behavioral symptoms at T2 |
| Behavioral symptoms | T3 | 317 | 0-18 | 4.1 (3.5) | Total behavioral symptoms at T3 |
| Temperament- Easy-difficult trait | T4 | 315 | -3.1- 2.1 | 0.02 (1) | Temperament factor reflecting distractibility/ soothability, mood, approach/withdrawal, and adaptability domains |
| Temperament- Self-control | T4 | 315 | -3.9 -1.9 | 0.1 (0.9) | Temperament factor reflecting attention/ persistence and distractibility/soothability domains |

| Function level-Family | T4 | 316 | 2-7 | 4.6 (0.9) | Evaluation of child functioning in the family |
| --- | --- | --- | --- | --- | --- |
| Function level- Peers | T4 | 316 | 2-7 | 4.6 (0.9) | Evaluation of peer relationships (e.g., popularity, position in the hierarchy) |
| Function level-Interests | T4 | 316 | 2-7 | 4.7 (0.9) | Evaluation of diversity of interets and free time activities |
| Function level-Autonomy | T4 | 316 | 2-7 | 4.7 (1) | Evaluation of mastery in developmentally assigned tasks |
| Self-perception-Scholastic competence | T4 | 315 | 8-24 | 19.3 (2.9) | Children’s perception of their academic and school performance |
| Self-perception-Social competence | T4 | 315 | 7-24 | 18.3 (3.3) | Children’s perception about their social skills and peer relationships |
| Self-perception-Athletic competence | T4 | 315 | 12-24 | 20.3 (2.5) | Children’s perception of physical ability and performance in sports or outdoor games |

T1: 3 months, T2:2 years, T3:4.5 years, T4:8 years, T6: 15 years. Polygenic scores were included in the first feature set since they reflect genetic propensity for diseases or traits independent from measurement time.

Model performance was comparable with the main analyses. For externalizing symptom trajectories, random forest achieved fair discriminatory power (Figure S11A), with a multiclass macro-average AUC of 0.70 (SD=0.08; 95% CI [0.65, 0.75]) and a multiclass micro-average of

0.87 (SD=0.04; 95% CI [0.84, 0.89]). Trajectory classes were classified with fair discriminatory

power (low symptoms: AUC = 0.70, SD = 0.12, 95% CI [0.64, 0.78]; increasing symptoms:

AUC = 0.64, SD = 0.15, 95% CI [0.55, 0.74]; decreasing symptoms: AUC = 0.76, SD = 0.15,

95% CI [0.67, 0.84]).

For internalizing symptom trajectories, support vector classifier achieved the highest performance (micro AUC: 0.90; macro AUC: 0.70; class-specific AUC: 0.65-0.77). However, given that model performance was comparable across classifiers, we report results from the logistic regression model to ensure consistency across analyses and to facilitate the interpretation of feature importance. Logistic regression achieved acceptable discriminatory power (Figure S11B), with a multiclass macro-average AUC of 0.63 (SD=0.14; 95% CI [0.55, 0.71]) and a multiclass micro-average of 0.80 (SD=0.09; 95% CI [0.74, 0.85]). All trajectory

classes were classified with above-chance level discriminatory power (low symptoms: AUC = 0.63, SD = 0.15, 95% CI [0.54, 0.71]; increasing symptoms: AUC = 0.61, SD = 0.15, 95% CI

[0.53, 0.70]; decreasing symptoms: AUC = 0.66, SD = 0.22, 95% CI [0.53, 0.79]).

**Figure S11.** Multinominal classification performance for externalizing (A) and internalizing

(B) symptom trajectories. Discriminatory accuracy over all trajectories and for each individual class were shown for each symptom domain. AUC indicates area under curve. Diagonal line represents chance-level performance.

# Trajectory Specific Features: Childhood Features Only

Shapley Additive Explations (SHAP) values were computed to reveal how much each feature contributes to class-specific model prediction (Figures S12-13). Features collected up to 8 years old (T4) were used in machine learning models.

**Figure S12.** Top 15 features contributing to class-specific model prediction for externalizing symptoms trajectories. Features on the Y-axis are ranked by mean absolute SHAP value, with higher positions indicating greater overall contribution to the model. The X-axis shows the distribution of SHAP values, where values to the right of zero indicate a positive contribution toward predicting the class and values to the left indicate a negative contribution. Color represents feature values, with blue indicating lower values and pink indicating higher values. Panels show low symptoms (A), increasing symptoms (B), and decreasing symptoms (C) trajectories. Abbreviations: ADHD, attention deficit and hyperactivity disorder; MDD, major depressive disorder;

NCEA, non-cognitive educational attainment; PGS, polygenic scores; SP, self-perception. Measurement times: T1= 3 months; T2= 2 years; T3= 4.5 years; T4= 8 years.

**Figure S13.** Top 15 features contributing to class-specific model prediction for internalizing symptoms trajectories. Features on the Y-axis are ranked by mean absolute SHAP value, with higher positions indicating greater overall contribution to the model. The X-axis shows the distribution of SHAP values, where values to the right of zero indicate a positive contribution toward predicting the class and values to the left indicate a negative contribution. Color represents feature values, with blue indicating lower values and pink indicating higher values. Panels show low symptoms (A), increasing symptoms (B), and decreasing symptoms (C) trajectories. Abbreviations: ADHD, attention deficit and hyperactivity disorder; MDD, major depressive disorder; NCEA, non-cognitive educational attainment; PGS, polygenic scores. Measurement times: T1= 3 months; T2= 2 years; T3= 4.5 years; T4= 8 years.

# Common Informant Bias

Common informant bias occurs when outcomes and predictors are derived from the same informant. In this study, internalizing and externalizing symptoms were used to model longitudinal symptom trajectories, and the resulting trajectory classes served as outcomes in the machine-learning analyses. Psychopathology symptoms were reported by parents at ages 8 and 11 and by participants from age 15 onward, while some predictors were also based on parent-and self-reports. To evaluate whether our findings were influenced by common informant bias, we repeated the machine-learning analyses using a separate feature set based on objective and quasi-objective measures (e.g., polygenic risk scores, hormone levels, observer-rated risk scores, standardized tests). The complete list of features is provided in Table S45.

**Table S45.** Feature list based on information source.

| Objective and Quasi-Objective Measures (n=43) | | Subjective Measures (n=30) | |
| --- | --- | --- | --- |
| Objective Measures (n=14) | Quasi-Objective Measures (n=29)  Observer-rated scores, diagnostics, standardized tests and metrics, demographics | Parent-Report (n=14) | Self-Report (n=16) |
| Biological sex at birth  Polygenic risk scores (n=10)  *ADHD, aggression, alcohol, anxiety, MDD, schizophrenia, cognitive performance, educational attainment, non-cognitive educational attainment, IQ*  Hormone levels (n=3)  *Testesterone, DHT, cortisol* | Obstetric risk Maternal stimulation  Adverse family factors (n=11)  *Low parental education, overcrowding, parental psychopathology, parental broken home, marital discord, one parent family, early parenthood, unwanted pregnancy, severe chronic life events, poor coping skills of a parent*  Temperament T4 (n=2) *Easy-difficult, self-control* Function levels T4 (n=4)  *Family, peers, interests, autonomy*  Function levels T5 (n=5)  *Family, peer, interests, school performance, autonomy*  Peer rejection  Parental alcohol dependence IQ (T5)  Income-to-need ratio (n=2)  *T1, T5* | Maternal smoking (n=2)  *Moderate, heavy*  Maternal stress during pregnancy  Maternal psychosomatic complaints  Stressful life events (n=6)  *T1 to T6*  Parental alcohol consumption  Behavioral symptoms of child (n=3)  *T1 to T3* | Bullying  Early alcohol onset Deviant peers  Childhood trauma (CTQ sum score)  Self-concept T4 (n=3)  *Cognition, peer, sport*  Temperament (n=4)  *Novelty, harm-avoidance, reward dependence, and persistence*  Self-Concept T6 (n=5) *Cognition, peer, physical competence, behaviorol conduct, self-value* |

For externalizing symptoms trajectories (Table S46), the best-performing model (Random Forest) showed performance comparable to the primary analysis (macro AUC = 0.74; class-specific AUCs = 0.70–0.81 vs. macro AUC=0.77 and class-specific AUCs=0.74–0.78, respectively). For internalizing symptoms trajectories (Table S47), performance was lower when relying solely on objective and quasi-objective measures (macro AUC = 0.67; class-specific AUCs = 0.62–0.74 vs macro AUC = 0.75; class-specific AUCs = 0.71–0.80).

**Table S46.** Model performance metrics for externalizing symptom trajectories across feature sets.

|  | Logistic Regression | Support Vector Classifier | Random Forest | XG Boost |
| --- | --- | --- | --- | --- |
| Objective/Quasi Objective | Micro AUC: 0.81  Macro AUC: 0.61 | Micro AUC: 0.89  Macro AUC: 0.60 | **Micro AUC: 0.88**  **Macro AUC: 0.74** | Micro AUC: 0.89  Macro AUC: 0.66 |
|  | Class-specific AUC | Class-specific AUC | **Class-specific AUC** | Class-specific AUC |
|  | Low: 0.63 | Low: 0.65 | **Low: 0.72** | Low: 0.64 |
|  | Increasing: 0.45 | Increasing: 0.40 | **Increasing: 0.70** | Increasing: 0.61 |
|  | Decreasing: 0.73 | Decreasing: 0.75 | **Decreasing: 0.81** | Decreasing: 0.72 |
| All | Micro AUC: 0.81 | Micro AUC: 0.90 | **Micro AUC: 0.88** | Micro AUC: 0.89 |
|  | Macro AUC: 0.64 | Macro AUC: 0.66 | **Macro AUC: 0.77** | Macro AUC: 0.67 |
|  | Class-specific AUC | Class-specific AUC | **Class-specific AUC** | Class-specific AUC |
|  | Low: 0.66 | Low: 0.66 | **Low: 0.74** | Low: 0.64 |
|  | Increasing: 0.64 | Increasing: 0.66 | **Increasing: 0.77** | Increasing: 0.69 |
|  | Decreasing: 0.64 | Decreasing: 0.67 | **Decreasing: 0.76** | Decreasing: 0.69 |

**Table S47.** Model performance metrics for internalizing symptom trajectories across feature sets.

|  | Logistic Regression | Support Vector Classifier | Random Forest | XG Boost |
| --- | --- | --- | --- | --- |
| Objective/Quasi Objective | **Micro AUC: 0.78**  **Macro AUC: 0.67** | Micro AUC: 0.91  Macro AUC: 0.72 | Micro AUC: 0.81  Macro AUC: 0.69 | Micro AUC: 0.88  Macro AUC: 0.66 |
|  | **Class-specific AUC** | Class-specific AUC | Class-specific AUC | Class-specific AUC |
|  | **Low: 0.65** | Low: 0.69 | Low: 0.67 | Low: 0.63 |
|  | **Increasing: 0.62** | Increasing: 0.65 | Increasing: 0.61 | Increasing: 0.62 |
|  | **Decreasing: 0.74** | Decreasing: 0.81 | Decreasing: 0.79 | Decreasing: 0.73 |
| All | **Micro AUC: 0.83** | Micro AUC: 0.91 | Micro AUC: 0.86 | Micro AUC: 0.86 |
|  | **Macro AUC: 0.75** | Macro AUC: 0.72 | Macro AUC: 0.75 | Macro AUC: 0.62 |
|  | **Class-specific AUC** | Class-specific AUC | Class-specific AUC | Class-specific AUC |
|  | **Low: 0.73** | Low: 0.71 | Low: 0.72 | Low: 0.60 |
|  | **Increasing: 0.70** | Increasing: 0.70 | Increasing: 0.69 | Increasing: 0.55 |
|  | **Decreasing: 0.80** | Decreasing: 0.76 | Decreasing: 0.82 | Decreasing: 0.72 |

# The Impact of Imputation Strategies

We used the IterativeImputer in Python to handle missing data in the machine-learning analyses. This multivariate imputation method estimates missing values by iteratively predicting each variable from the remaining predictors using regression-based models. To ensure that our findings were not influenced by the choice of imputation approach, we compared model performance across alternative strategies implemented in sklearn.impute, including mean imputation, median imputation, and k-nearest neighbors (KNN) imputation, while keeping all other aspects of the analytic pipeline identical to the primary analyses. Model performance was comparable across imputation methods (Tables S48–S49) and did not alter the interpretation of the results.

**Table S48.** Model performance metrics across different imputation techniques: externalizing symptoms trajectories.

|  | Logistic Regression | Support Vector Classifier | Random Forest | XG Boost |
| --- | --- | --- | --- | --- |
| Iterative Imputer | Micro AUC: 0.81 | Micro AUC: 0.90 | Micro AUC: 0.88 | Micro AUC: 0.89 |
|  | Macro AUC: 0.64 | Macro AUC: 0.66 | Macro AUC: 0.77 | Macro AUC: 0.67 |
|  | Class-specific AUC | Class-specific AUC | Class-specific AUC | Class-specific AUC |
|  | Low: 0.66 | Low: 0.66 | Low: 0.74 | Low: 0.64 |
|  | Increasing: 0.64 | Increasing: 0.66 | Increasing: 0.77 | Increasing: 0.69 |
|  | Decreasing: 0.64 | Decreasing: 0.67 | Decreasing: 0.76 | Decreasing: 0.69 |
| Mean | Micro AUC: 0.85 | Micro AUC: 0.89 | Micro AUC: 0.88 | Micro AUC: 0.89 |
|  | Macro AUC: 0.67 | Macro AUC: 0.66 | Macro AUC: 0.77 | Macro AUC: 0.67 |
|  | Class-specific AUC | Class-specific AUC | Class-specific AUC | Class-specific AUC |
|  | Low: 0.67 | Low: 0.65 | Low: 0.74 | Low: 0.65 |
|  | Increasing: 0.64 | Increasing: 0.65 | Increasing: 0.77 | Increasing: 0.72 |
|  | Decreasing: 0.69 | Decreasing: 0.67 | Decreasing: 0.80 | Decreasing: 0.70 |
| Median | Micro AUC: 0.82 | Micro AUC: 0.90 | Micro AUC: 0.88 | Micro AUC: 0.89 |
|  | Macro AUC: 0.64 | Macro AUC: 0.67 | Macro AUC: 0.74 | Macro AUC: 0.67 |
|  | Class-specific AUC | Class-specific AUC | Class-specific AUC | Class-specific AUC |
|  | Low: 0.66 | Low: 0.67 | Low: 0.73 | Low: 0.64 |
|  | Increasing: 0.61 | Increasing: 0.63 | Increasing: 0.71 | Increasing: 0.72 |
|  | Decreasing: 0.66 | Decreasing: 0.70 | Decreasing: 0.78 | Decreasing: 0.66 |
| KNN | Micro AUC: 0.82 | Micro AUC: 0.90 | Micro AUC: 0.87 | Micro AUC: 0.89 |
|  | Macro AUC: 0.66 | Macro AUC: 0.68 | Macro AUC: 0.73 | Macro AUC: 0.67 |
|  | Class-specific AUC | Class-specific AUC | Class-specific AUC | Class-specific AUC |
|  | Low: 0.67 | Low: 0.67 | Low: 0.72 | Low: 0.63 |
|  | Increasing: 0.65 | Increasing: 0.66 | Increasing: 0.70 | Increasing: 0.74 |
|  | Decreasing: 0.67 | Decreasing: 0.70 | Decreasing: 0.76 | Decreasing: 0.65 |

**Table 49.** Model performance metrics across different imputation techniques: internalizing symptoms trajectories.

|  | Logistic Regression | Support Vector Classifier | Random Forest | XG Boost |
| --- | --- | --- | --- | --- |
| Iterative Imputer | Micro AUC: 0.83 | Micro AUC: 0.91 | Micro AUC: 0.86 | Micro AUC: 0.86 |
|  | Macro AUC: 0.75 | Macro AUC: 0.72 | Macro AUC: 0.75 | Macro AUC: 0.62 |
|  | Class-specific AUC | Class-specific AUC | Class-specific AUC | Class-specific AUC |
|  | Low: 0.73 | Low: 0.71 | Low: 0.72 | Low: 0.60 |
|  | Increasing: 0.70 | Increasing: 0.70 | Increasing: 0.69 | Increasing: 0.55 |
|  | Decreasing: 0.80 | Decreasing: 0.76 | Decreasing: 0.82 | Decreasing: 0.72 |
| Mean | Micro AUC: 0.82 | Micro AUC: 0.90 | Micro AUC: 0.88 | Micro AUC: 0.88 |
|  | Macro AUC: 0.74 | Macro AUC: 0.71 | Macro AUC: 0.75 | Macro AUC: 0.67 |
|  | Class-specific AUC | Class-specific AUC | Class-specific AUC | Class-specific AUC |
|  | Low: 0.72 | Low: 0.70 | Low: 0.72 | Low: 0.64 |
|  | Increasing: 0.71 | Increasing: 0.67 | Increasing: 0.72 | Increasing: 0.62 |
|  | Decreasing: 0.79 | Decreasing: 0.75 | Decreasing: 0.82 | Decreasing: 0.74 |
| Median | Micro AUC: 0.81 | Micro AUC: 0.90 | Micro AUC: 0.88 | Micro AUC: 0.88 |
|  | Macro AUC: 0.74 | Macro AUC: 0.71 | Macro AUC: 0.76 | Macro AUC: 0.66 |
|  | Class-specific AUC | Class-specific AUC | Class-specific AUC | Class-specific AUC |
|  | Low: 0.74 | Low: 0.71 | Low: 0.72 | Low: 0.64 |
|  | Increasing: 0.70 | Increasing: 0.67 | Increasing: 0.74 | Increasing: 0.61 |
|  | Decreasing: 0.79 | Decreasing: 0.76 | Decreasing: 0.82 | Decreasing: 0.73 |
| KNN | Micro AUC: 0.82 | Micro AUC: 0.90 | Micro AUC: 0.88 | Micro AUC: 0.89 |
|  | Macro AUC: 0.73 | Macro AUC: 0.72 | Macro AUC: 0.76 | Macro AUC: 0.70 |
|  | Class-specific AUC | Class-specific AUC | Class-specific AUC | Class-specific AUC |
|  | Low: 0.70 | Low: 0.70 | Low: 0.73 | Low: 0.66 |
|  | Increasing: 0.69 | Increasing: 0.69 | Increasing: 072 | Increasing: 0.66 |
|  | Decreasing: 0.79 | Decreasing: 0.76 | Decreasing: 0.83 | Decreasing: 0.75 |

# References

1. Achenbach TM. Integrative Guide for the 1991 CBCL/4-18, YSR, and TRF Profiles. Burlington, VT, USA: University of Vermont Department of Psychiatry; 1991.
2. Berg-Nielsen TS, Vika A, Dahl AA. When adolescents disagree with their mother: CBCL-YSR discrepancies related to maternal depression and adolescent self-esteem. Child Care Health Dev. 2003;29(3).
3. Chen YY, Ho SY, Lee PC, Wu CK, Gau SSF. Parent-child discrepancies in the report of adolescent emotional and behavioral problems in Taiwan. PLoS ONE. 2017;12(6).
4. Rana M, Delker E, East P, Blanco E, Burrows R, Lozoff B, et al. Mother-Adolescent Discrepancies in Ratings of Adolescents’ Adjustment: Associations with Maternal Mental Health and Family Factors. J Dev Behav Pediatr [Internet]. 2021 Apr [cited 2026 Jan 18];42(3):198–204. Available from: <https://journals.lww.com/10.1097/DBP.0000000000000879>
5. Laucht M, Esser G, Baving L, Gerhold M, Hoesch I, Ihle W, et al. Behavioral sequelae of perinatal insults and early family adversity at 8 years of age. J Am Acad Child Adolesc Psychiatry. 2000;39(10):1229–37.
6. Monninger M, Aggensteiner PM, Pollok TM, Reinhard I, Hall ASM, Zillich L, et al. Real-time individual benefit from social interactions before and during the lockdown: the crucial role of personality, neurobiology and genes. Transl Psychiatry. 2022;12(1).
7. Chang CC, Chow CC, Tellier LCAM, Vattikuti S, Purcell SM, Lee JJ. Second-generation PLINK: Rising to the challenge of larger and richer datasets. GigaScience. 2015;4(1).
8. Demontis D, Walters RK, Martin J, Mattheisen M, Als TD, Agerbo E, et al. Discovery of the first genome-wide significant risk loci for attention deficit/hyperactivity disorder. Nat Genet. 2019;51(1).
9. Pappa I, St Pourcain B, Benke K, Cavadino A, Hakulinen C, Nivard MG, et al. A genome-wide approach to children’s aggressive behavior: *The EAGLE consortium*. Am J Med Genet B Neuropsychiatr Genet [Internet]. 2016 July [cited 2026 Jan 18];171(5):562–

72. Available from: <https://onlinelibrary.wiley.com/doi/10.1002/ajmg.b.32333>

10. Walters RK, Polimanti R, Johnson EC, McClintick JN, Adams MJ, Adkins AE, et al. Transancestral GWAS of alcohol dependence reveals common genetic underpinnings with psychiatric disorders. Nat Neurosci. 2018;21(12).
11. Purves K, Coleman J, Gaspar HA, Breen G, Eley T. Genome-Wide Association Study of Generalized Anxiety Disorder Symptoms and Self-Reported Anxiety Disorder: An Investigation of Unique and Shared Genomic Contributions. Eur Neuropsychopharmacol. 2019;29.
12. Lee JJ, Wedow R, Okbay A, Kong E, Maghzian O, Zacher M, et al. Gene discovery and polygenic prediction from a genome-wide association study of educational attainment in
    12.1 million individuals. Nat Genet. 2018;50(8).
13. Savage JE, Jansen PR, Stringer S, Watanabe K, Bryois J, De Leeuw CA, et al. Genome-wide association meta-analysis in 269,867 individuals identifies new genetic and functional links to intelligence. Nat Genet. 2018;50(7).
14. Demange PA, Malanchini M, Mallard TT, Biroli P, Cox SR, Grotzinger AD, et al. Investigating the genetic architecture of noncognitive skills using GWAS-by-subtraction. Nat Genet. 2021;53(1).
15. Howard DM, Adams MJ, Shirali M, Clarke TK, Marioni RE, Davies G, et al. Genome-wide association study of depression phenotypes in UK Biobank identifies variants in excitatory synaptic pathways. Nat Commun. 2018;9(1).
16. Ripke S, Neale BM, Corvin A, Walters JTR, Farh KH, Holmans PA, et al. Biological insights from 108 schizophrenia-associated genetic loci. Nature. 2014;511(7510).
17. Choi SW, O’Reilly PF. PRSice-2: Polygenic Risk Score software for biobank-scale data. GigaScience. 2019;8(7).
18. Schultebraucks K, Choi KW, Galatzer-Levy IR, Bonanno GA. Discriminating Heterogeneous Trajectories of Resilience and Depression after Major Life Stressors Using Polygenic Scores. JAMA Psychiatry. 2021;78(7).
19. Poustka L, Maras A, Hohm E, Fellinger J, Holtmann M, Banaschewski T, et al. Negative association between plasma cortisol levels and aggression in a high-risk community sample of adolescents. J Neural Transm. 2010;117(5).
20. Maras A, Laucht M, Gerdes D, Wilhelm C, Lewicka S, Haack D, et al. Association of testosterone and dihydrotestosterone with externalizing behavior in adolescent boys and girls. Psychoneuroendocrinology. 2003;28(7).
21. Holz NE, Boecker R, Baumeister S, Hohm E, Zohsel K, Buchmann AF, et al. Effect of Prenatal Exposure to Tobacco Smoke on Inhibitory Control Neuroimaging Results from a 25-Year Prospective Study. JAMA Psychiatry. 2014;71(7):786–96.
22. Zohsel K, Buchmann AF, Blomeyer D, Hohm E, Schmidt MH, Esser G, et al. Mothers’ prenatal stress and their children’s antisocial outcomes - A moderating role for the Dopamine D4 Receptor (DRD4) gene. J Child Psychol Psychiatry. 2014;55(1):69–76.
23. Rice F, Harold GT, Boivin J, Van Den Bree M, Hay DF, Thapar A. The links between prenatal stress and offspring development and psychopathology: Disentangling environmental and inherited influences. Psychol Med. 2010;40(2):335–42.
24. Zerssen D von, Koeller DM. Die Beschwerden-Liste. Weinheim: Beltz; 1976.
25. Berth, Hendrick, Balck, Friedrich. Psychologische Tests für Mediziner. Springer; 2003.
26. Holz NE, Boecker-Schlier R, Jennen-Steinmetz C, Hohm E, Buchmann AF, Blomeyer D, et al. Early maternal care may counteract familial liability for psychopathology in the reward circuitry. Soc Cogn Affect Neurosci. 2018;13(11):1191–201.
27. Esser, G., Scheven, A., Petrova, A., Laucht, M., Schmidt, M.H. Mannheimer Beurteilungsskala zur Erfassung der Mutter-Kind-Interaktion im Säuglingsalter (MBS-MKI-S). 1989;17:185–93.
28. Esser G, Laucht M, Schmidt M, Löffler W, Reiser A, Stöhr RM, et al. Behaviour problems and developmental status of 3-month-old infants in relation to organic and psychosocial risks. Eur Arch Psychiatry Neurol Sci [Internet]. 1990 Nov [cited 2026 Jan 19];239(6):384–90. Available from: <http://link.springer.com/10.1007/BF01734547>
29. OECD. OECD list of social indicators. Paris: OECD Publications and Information Center; 1982.
30. Wittchen HU, Wunderlich U, Gruschwitz S, Zaudig M. SKID I. Strukturiertes Klinisches Interview für DSM-IV. Achse I: Psychische Störungen. Interviewheft und Beurteilungsheft. Eine deutschsprachige, erweiterte Bearb. d. amerikanischen Originalversion des SKID I. Goettingen: Hogrefe; 1997.
31. Marcus A, Blanz B, Esser G, Niemeyer J, Schmidt MH. Beurteilung des Funktionsniveaus bei Kindern und Jugendlichen mit psychischen Störungen. Kinderheit Entwickl. 1993;2:166–72.
32. Marcus, A., Blanz, B., Esser, G., Niemeyer, J., Schmidt, M.H. Beurteilung des Funktionsniveaus bei Kindern und Jugendlichen mit psychischen Stoerungen. 1993;2:166–72.
33. Dawson DA, Goldstein RB, Patricia Chou S, June Ruan W, Grant BF. Age at first drink and the first incidence of adult-onset DSM-IV alcohol use disorders. Alcohol Clin Exp Res. 2008;32(12).
34. Bernstein DP, Fink L, Handelsman L, Foote J, Lovejoy M, Wenzel K, et al. Initial reliability and validity of a new retrospective measure of child abuse and neglect. Am J Psychiatry. 1994;151(8).
35. Wingenfeld K, Hill A, Gast U, Beblo T, Höpp H, Schlosser N, et al. Die deutsche Version des Childhood Trauma Questionnaire (CTQ): Erste Befunde zu den psychometrischen Kennwerten. PPmP - Psychother · Psychosom · Med Psychol. 2010;60(11):442–50.
36. Maier-Diewald W, Wittchen HU, Hecht H, Werner-Eilert K. Die Münchner Ereignisliste (MEL) - Anwendungsmanual. München; 1983.
37. Wittchen HU, Ahmoi Essau C, Hecht H, Teder W, Pfister H. Reliability of life event assessments: test—retest reliability and fall-off effects of the Munich interview for the assessment of life events and conditions. J Affect Disord [Internet]. 1989 Jan [cited 2026 Jan 19];16(1):77–91. Available from: <https://linkinghub.elsevier.com/retrieve/pii/0165032789900591>
38. Laucht M, Esser G, Schmidt MH. Developmental outcome of infants born with biological and psychosocial risks. J Child Psychol Psychiatry. 1997;38(7).
39. Thomas A, Chess A, Birch HG. Temperamentand behaviour disorders in children. New York: New York University Press; 1968.
40. Weiss R. Grundintelligenztest CFT 20. Göttingen: Hogrefe; 1987.
41. Weiss R. Wortschatztest WS. Ergänzungstest zumGrundintelligenztest CFT 20. Göttingen: Hogrefe; 1987.
42. Pitzer M, Esser G, Schmidt MH, Laucht M. Temperament in the developmental course: a longitudinal comparison of New York Longitudinal Study-derived dimensions with the Junior Temperament and Character Inventory. Compr Psychiatry. 2007;48(6).
43. Pitzer M, Esser G, Schmidt MH, Laucht M. Temperamental predictors of externalizing problems among boys and girls: A longitudinal study in a high-risk sample from ages 3 months to 15 years. Eur Arch Psychiatry Clin Neurosci. 2009;259(8).
44. Schmeck K, Goth K, Poustka F, Cloninger RC. Reliability and validity of the Junior Temperament and Character Inventory. Int J Methods Psychiatr Res. 2001;10(4).
45. Asendorpf JB, Van Aken MAG. Deutsche Versionen der Selbstkonzeptskalen von Harter. Pädagog Psychol Band XXV. 1993;1.
46. Babor TF, Grant M. From clinical research to secondary prevention: international collaboration in the development of the Alcohol Disorders Identification Test (AUDIT). 1989. (Alcohol Health and Research World; vol. 13).
47. Dybek I, Bischof G, Grothues J, Reinhardt S, Meyer C, Hapke U, et al. The Reliability and Validity of the Alcohol Use Disorders Identification Test (AUDIT) in a German General Practice Population Sample. J Stud Alcohol [Internet]. 2006 May [cited 2026 Jan 19];67(3):473–81. Available from: <https://www.jsad.com/doi/10.15288/jsa.2006.67.473>
48. Janke W, Erdmann G. SVF 78: Eine Kurzform des Stressverarbeitungsfragebogens SVF

120. Göttingen: Hogrefe; 2002.

49. Ising M, Weyers P, Janke W, Erdmann G. Die Gütekriterien des SVF78 von Janke und Erdmann, einer Kurzform des SVF120. Z Für Differ Diagn Psychol [Internet]. 2001 Dec [cited 2026 Jan 19];22(4):279–89. Available from: <https://econtent.hogrefe.com/doi/10.1024//0170-1789.22.4.279>
50. Hampel P, Dickow B, Petermann F. Reliabilität und Validität des SVF-KJ. Z Für Differ Diagn Psychol [Internet]. 2002 Sept [cited 2026 Jan 19];23(3):273–89. Available from: <https://econtent.hogrefe.com/doi/10.1024//0170-1789.23.3.273>
51. Holz NE, Boecker R, Jennen-Steinmetz C, Buchmann AF, Blomeyer D, Baumeister S, et al. Positive coping styles and perigenual ACC volume: Two related mechanisms for conferring resilience? Soc Cogn Affect Neurosci. 2016;
52. Dick A. Psychotherapie und Glück: Quellen und Prozesse seelischer Gesundheit. Bern: Huber; 2003.
